# Dynamic distribution of systemically administered antibiotics in orthopeadically relevant target tissues and settings

**DOI:** 10.1111/apm.13490

**Published:** 2024-11-12

**Authors:** Maria Bech Damsgaard Nielsen, Andrea René Jørgensen, Maiken Stilling, Mads Kristian Duborg Mikkelsen, Nis Pedersen Jørgensen, Mats Bue

**Affiliations:** ^1^ Department of Clinical Medicine Aarhus University Aarhus N Denmark; ^2^ Aarhus Denmark Microdialysis Research (ADMIRE) Orthopaedic Research Laboratory, Aarhus University Hospital Aarhus N Denmark; ^3^ Department of Orthopaedic Surgery Aarhus University Hospital Aarhus N Denmark; ^4^ Department of Infectious Diseases Aarhus University Hospital Aarhus N Denmark

**Keywords:** Antibiotics, orthopaedic, pharmacokinetics, penetration, bone

## Abstract

This review aimed to summarize the current literature on antibiotic distribution in orthopedically relevant tissues and settings where dynamic sampling methods have been used. PubMed and Embase databases were systematically searched. English‐published studies between 2004 and 2024 involving systemic antibiotic administration in orthopedically relevant tissues and settings based on dynamic measurements were included. In total, 5385 titles were identified. After title and abstract screening, 97 eligible studies (43 different antibiotic drugs) were included. The studies covered both preclinical (42%) and clinical studies including healthy and infected tissues (21%) and prophylactic and steady‐state situations (35%). Microdialysis emerged as the predominant sampling method in 98% of the studies. Most of the presented antibiotics (80%) were only assessed once or twice. Among the most extensively studied antibiotics were cefuroxime (18 studies), linezolid (9 studies) and vancomycin (9 studies). This review presents valuable insights into the microenvironmental distribution of antibiotics in orthopedically relevant target tissues and settings and seeks to provide a basis for improving dosing recommendations and treatment outcomes. However, it is important to acknowledge that our findings are limited to the specific drug, dosing regimens, administration method and target tissue, and are crucially linked to the selected PK/PD target.

## INTRODUCTION

Orthopaedic infections, including periprosthetic joint infections (PJI), fracture‐related infections (FRI), osteomyelitis, bacterial arthritis and spondylodiscitis, are heterogenous conditions that are notoriously complicated to treat [1, 2]. The morbidity is significant, as patients often experience pain, reduced functional outcomes, prolonged hospitalization, numerous surgical procedures and risk of amputation, leading to poor quality of life [3, 4, 5]. The treatment is costly for both the patient and the healthcare system [6, 7, 8], and mortality rates are high [5, 9, 10]. For example, 5 years survival rates for PJI have been found to be comparable with common cancers like melanoma, breast cancer and prostate cancer [10], and the direct healthcare costs of single‐occurrence FRI patients are three times higher as compared to non‐FRI patients [7]. Systemic antibiotic administration is an essential tool in preventing and treating orthopaedic infections, which is why measures to understand and improve antibiotic administration regimens are critical [11, 12, 13, 14].

Optimal antibiotic treatment entails sufficient target site exposure. Current knowledge of antibiotic tissue distribution following common routes of systemic administration to orthopedically relevant target tissues and settings is limited. Three reviews of antibiotic bone penetration have been performed, primarily reporting data from bone biopsies or tissue specimens [15, 16, 17]. This methodology does not provide a temporal resolution, which precludes any evaluation of a dynamic pharmacokinetic/pharmacodynamic (PK/PD) target. Moreover, tissue specimens must be homogenized before analysis, allowing for only the total antibiotic concentrations to be determined [18]. As the unbound interstitial fraction of an antibiotic is considered to be the microbiologically active, this further challenges the applicability of these findings [19]. Two reviews have evaluated the available dynamic knowledge of interstitial fluid concentrations from several target tissues and plasma but without an orthopaedic focus [20, 21]. A recent review described the existing microdialysis studies of different antibiotics in muscle and subcutaneous tissue (SCT) but did not include bone or joints [22].

Antibiotic target tissue distribution cannot be generalized and must consider the specific clinical scenario and setting. For example, several studies across tissues and drugs have displayed heterogeneous tissue distribution [23, 24, 25, 26, 27, 28, 29], and the sufficiency of exposure is conditioned by the clinical setting (e.g. prophylactic vs. therapeutic situations). Furthermore, drug‐specific PK/PD knowledge is needed for determining relevant systemic dosing intervals and regimens. This review aimed to summarize the current literature on antibiotic distribution in orthopedically relevant tissues and settings based on dynamic sampling methods.

## METHODS

### Information sources

A literature search was conducted with the assistance of a scientific librarian from Aarhus University. PubMed and Embase databases were systematically searched using relevant keywords, Emtrees and MeSH terms, such as anti‐bacterial agents, anti‐infective agents, antibiotics, antimicrobials, pharmacokinetics, tissue distribution, bone, bones, synovial fluid, synovial fluids, joints and connective tissue. Pharmacokinetics was searched as a MeSH term subheading. Several hits were found using truncated keywords or quotation marks but only searched in the title or abstract to further narrow/specify the results. The search was limited to publications after 2004 as Ultra High‐Performance Liquid Chromatography (UHPLC) as an antibiotic concentration quantification tool was first introduced in 2004 [30]. Finally, citation analysis and reference search were conducted on the included studies. Some of these references were not available in either of the databases but were found and included from Google Scholar.

The search string was last performed on 14.08.2024. Details of the protocol for this systematic review were registered on PROSPERO and can be accessed at:

https://www.crd.york.ac.uk/prospero/display_record.php?RecordID=462637

### Eligibility criteria and study selection

Article selection was performed in accordance with the PRISMA guidelines [31]. First, a minimum of two reviewers screened titles and abstracts for relevance using Covidence [32]. Second, two reviewers independently screened full texts and selected them based on the inclusion and exclusion criteria. Any disagreements were resolved by screening by a third reviewer.

Exclusion criteria

1. Local antibiotic administration
2. Patients aged <18 in clinical studies
3. No temporal resolution in the measurements
4. Data based on biopsies or other tissue specimen methods providing only a total concentration
5. Non‐original articles
6. Publications from before 2004
7. Editorials or conference abstracts
8. Cadaver studies

### Data collection process

Two reviewers extracted data from the text, tables and supplements. Irrespective of study settings, data regarding dynamic antibiotic concentrations from orthopedically relevant tissues in vivo were extracted. The PK/PD parameters included time above minimum inhibitory concentration (T >MIC), peak drug concentration (Cmax) and tissue penetration.

## RESULTS

### Retrieved articles

**Fig. 1:**
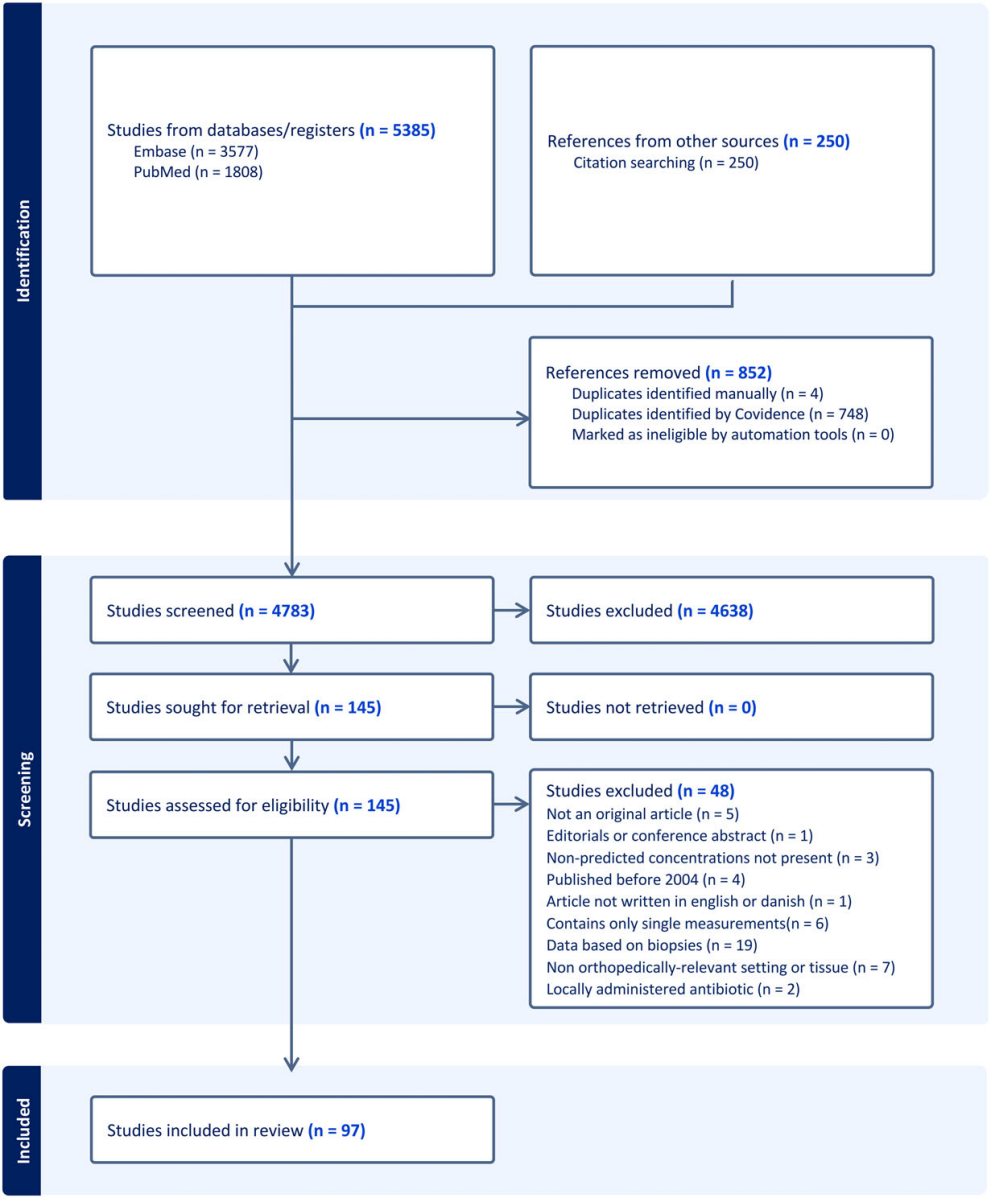
PRISMA Flowchart of study selection [31].

Fig. 1 provides the flowchart of the study selection. In total, 5385 records were identified from the search string, and 250 references were found from citation searching. A total of 852 references were marked as duplicates, leaving 4783 to be screened by title and abstract. In total, 145 articles were chosen for retrieval, and all were available. Based on the exclusion criteria, 48 articles were excluded, and 97 studies were deemed eligible for data extraction.

### Main findings

#### Study populations

**Fig. 2:**
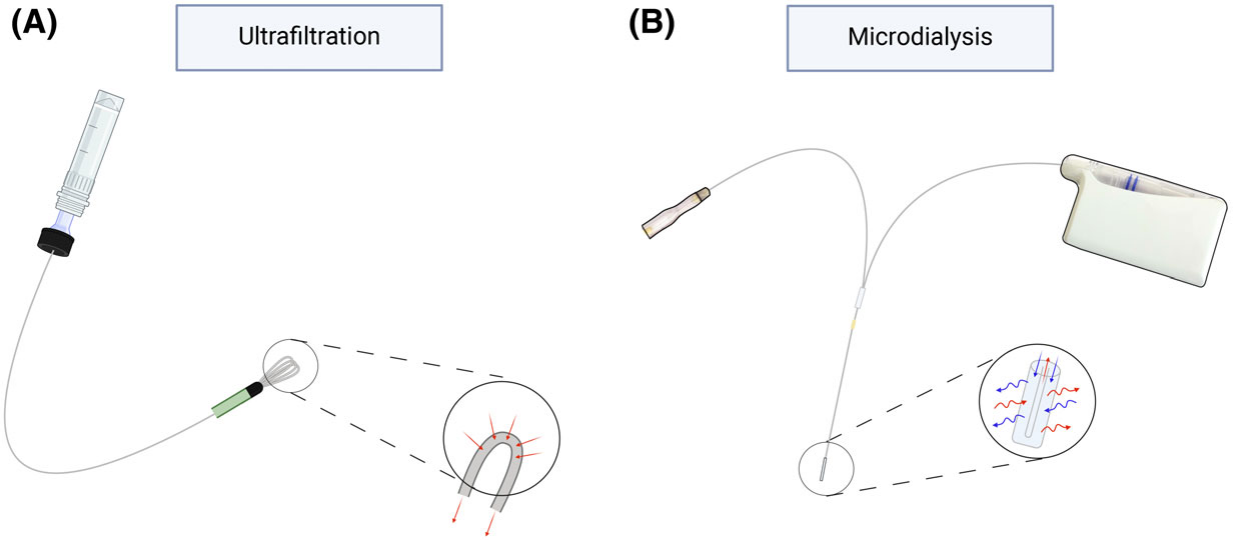
(A) Ultrafiltration relies on vacuum as a driving force to extract interstitial fluid (*n* = 2/97 studies). (B) Microdialysis relies on continuous concentration‐driven diffusion (*n* = 95/97 studies).

Most of the studies were performed on healthy volunteers, diabetic foot patients or pigs. Some were performed on rats, rabbits and horses. The target tissues were SCT, muscle, bone (cancellous, cortical and the medullary canal), intervertebral disc and synovial fluid. Ultrafiltration and microdialysis (Fig. 2) were used as dynamic sampling methods.

### Number of studies with dynamic measurements

**Fig. 3:**
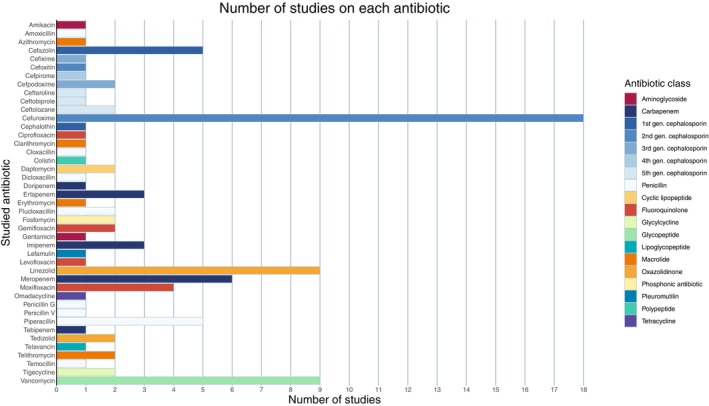
Graphical presentation of the number of studies according to antibiotic drug.

The majority of antibiotics have only been studied once or twice in a dynamic setting (Fig. 3). Cefuroxime, vancomycin and linezolid were among the most investigated antibiotics.

### Individual antibiotic distribution in orthopeadically relevant tissues and settings

The results have been grouped by drug type and presented alphabetically. Table 1 summarizes the different studies' study groups, dosing and applied PK/PD targets. The following text provides an outline of each study's results and conclusions.

**Table 1:** An overview of antibiotic distibution in orthopeadically relevant tissues and settings.

Study	Antibiotic	Study group	Dosage and setting	Plasma T >MIC	Tissue T >MIC	Plasma Cmax	Tissue Cmax	Tissue penetration
Aminoglycoside								
Pinto 2011, USA [33]	Amikacin	Horses (*n* = 6), Weight: 517 kg	10 mg/kg, IV‐bolus, Prophylactic (24 h sampling)				**SCT** (neck): 12.7 ug/mL **Synovial fluid** (radiocarpal joint): 19.7 ug/mL	
Stolle 2004, Denmark [34]	Gentamicin	Pigs (*n* = 8), Weight range: 42–48 kg	240 mg, IV‐bolus, Prophylactic (6.5 h sampling)			**Total plasma**: 33 mg/L	**Cancellous bone** (medial tibia): 6.7 mg/L **Cancellous bone** (lateral tibia): 6.5 mg/L	**Cancellous bone** (medial tibia): 0.48 **Cancellous bone** lateral tibia: 0.54
**Beta Lactams**								
Carbapenem								
Burian 2012, Austria [35]	Doripenem	Healthy volunteers (*n* = 6 M), Age: 30 years, BMI: 22.9, CrCl: 108 mL/min	500 mg, IV‐bolus, Prophylactic (8 h sampling)		**Skeletal muscle** (thigh): MIC 1.5 mg/L: <40% **SCT** (thigh): MIC 2 mg/L: >40%	**Total plasma**: 15.3 mg/L	**Skeletal muscle** (thigh): 6.6 mg/L **SCT** (thigh): 9.9 mg/L	
Mangum 2019, USA [37]	Ertapenem^1^	Rats, Sprague–Dawley (*n* = 9)	35 mg/kg, IV‐bolus, Prophylactic (72 h sampling)			**Free plasma**: 135 ug/mL	**Skeletal muscle** (hind leg) non‐ischemic limb: 18 ug/mL	**Skeletal muscle** (hind leg) non‐ischemic limb: 0.10
Burkhardt 2006, USA [36]	Ertapenem	Healthy volunteers (*n* = 6, 3F 3M), Age range: 22–37, BMI: 25.3, CrCl: 100.5 mL/min^1^ 1.73 m^2	1 g, IV‐bolus, Prophylactic (12 h sampling)		**Skeletal muscle** (thigh): MIC 4 mg/L <50% **SCT** (thigh): MIC 4 mg/L >30%	**Total plasma**: 103.3 mg/L	**Skeletal muscle** (thigh): 6.71 mg/L **SCT** (thigh): 3.96 mg/L	**Skeletal muscle** (thigh): 0.13 **SCT** (thigh): 0.05
Sauermann 2013, Austria [38]	Ertapenem	Patient with DFI (*n* = 9), Age: 55, BMI: 33.6, CrCl: 1.2 mg/dL	1 g daily x 3 days, IV‐bolus, steady state (8 h sampling)		**SCT** (DFI): MIC 1 mg/L: 38%	**Total plasma**: 59.5 mg/L	**SCT** (thigh): 2.4 mg/L **SCT** (DFI): 4.5 mg/L	
Dahyot 2006, France [39]	Imipenem	Rats, Acinetobacter baumannii lung infection (*n* = 7), Weight: 331 g	30 mg/kg, IV‐bolus, therapeutic (1.5 h sampling)			**Free plasma**: 55.6 ug/mL	**Skeletal muscle** (hind leg): 53.9 ug/mL	**Skeletal muscle** (hind leg): 0.95
Marchand 2005, France [40]	Imipenem	Rats (*n* = 10), Weight: 288 g Hydrated (*n* = 6), non‐hydrated (*n* = 4)	120 mg/kg, IV‐bolus, prophylactic (2.5 h sampling)			**Free plasma**: Hydrated: 226 ug/mL Non‐hydrated: 253 ug/mL	**Skeletal muscle** (hind leg): Hydrated: 228 ug/mL Non‐hydrated: 292 ug/ml	**Skeletal muscle** (hind leg): Hydrated: 1.02 Non‐hydrated: 1.16
Marchand 2005, France [41]	Imipenem	Rats (*n* = 16), Weight: 294 g, Hypovolemic (*n* = 8), Normovolemic (8)	70 mg/kg, IV‐bolus, prophylactic (2 h sampling)			**Free plasma**: Hypovolemic: 132 ug/mL Normovolemic: 115 ug/mL	**Skeletal muscle** (hind leg): Hypovolemic: 125 ug/mL Normovolemic: 111 ug/mL	**Skeletal muscle** (hind leg): Hypovolemic: 1.00 Normovolemic: 0.97
Hanberg 2019, Denmark [42]	Meropenem	Pigs (*n* = 6), Weight range: 72–81 kg	1000 mg, IV‐bolus, Prophylactic (8 h sampling)			**Free plasma**: 74.3 ug/mL	**Cancellous bone** (tibia): 21.4 ug/mL **SCT** (thoracic wall): 81.6	**Cancellous bone** (tibia): 0.56 **SCT** (thoracic wall): 1.5
Slater 2023, Denmark [43]	Meropenem^3^	Pigs (*n* = 8), Weight range: 78–82 kg	1000 mg, IV‐bolus, Prophylactic (8 h sampling)	**Free plasma**: MIC 0.125–2 ug/mL: 406–181 min, 90%–40%	**Cancellous bone** (C3): MIC 0.125–2 ug/mL: 244–114 min, 54%–25% **Paravertebral skeletal muscle**: MIC 0.125–2 ug/mL: 285–118 min, 63%–26 % **SCT** (neck): MIC 0.125–2 ug/mL: 334–163 min, 74%–36 % **Intervertebral disc** (C3‐C4): MIC 0.125–2 ug/mL: 366–158 min, 81%–35%	**Free plasma**: 67 ug/mL	**Cancellous bone** (C3): 9 ug/mL **Paravertebral skeletal muscle**: 10 ug/mL **SCT** (neck): 14 ug/mL **Intervertebral disc** (C3‐C4): 5 ug/mL	**Cancellous bone** (C3): 0.24 **Paravertebral skeletal muscle**: 0.27 **SCT** (neck): 0.42 **Intervertebral disc** (C3‐C4): 0.21
Vittrup 2022, Denmark [44]	Meropenem^3^	Pigs (*n* = 8), Weight range: 78–82 kg	1000 mg, IV‐bolus, Prophylactic (8 h sampling)	**Free plasma**: MIC 0.125–2 ug/mL: 406–181 min, 90%–40%	**Cancellous bone** (tibia): MIC 0.125–2 ug/mL: 278–144 min, 62%–32% **Cortical bone** (tibia): MIC 0.125–2 ug/mL: 189–45 min, 42%–10% **SCT** (tibia): MIC 0.125–2 ug/mL: 331–180, 74%–40%	**Free plasma**: 67 ug/mL	**Cancellous bone** (tibia): 14 ug/mL **Cortical bone** (tibia): 3 ug/mL **SCT** (tibia): 22 ug/mL	
Vittrup 2022, Denmark [45]	Meropenem^3^	Pigs (*n* = 8), Weight range: 78–82 kg	1000 mg, IV‐bolus, Prophylactic (8 h sampling)	**Free plasma**: MIC 0.125–2 ug/mL: 406–181 min, 90%–40%	**Cannulated screw** (tibia): MIC 0.125–2 ug/mL: 148–17 min, 33%–4% **Cancellous bone adjacent to screw** (tibia): MIC 0.125–2 ug/mL: 278–133 min, 62%–29% **Cancellous bone on contralateral leg** (tibia): MIC 0.125–2 ug/mL: 278–144 min, 62%–32%	**Free plasma**: 67 ug/mL	**Cannulated screw** (tibia): 1 ug/mL **Cancellous bone adjacent to screw** (tibia): 9 ug/mL **Cancellous bone on contralateral leg** (tibia): 14 ug/mL	
Simon 2020, Germany [46]	Meropenem^2^	Obese surgical patients (*n* = 2M13F), Age: 50.3, BMI: 48.7, S‐Cr:85.8 umol/L. Non‐obese patients (*n* = 2M13F), Age: 49.5, BMI: 23.9, S‐Cr: 75.3 umol/L	1000 mg, IV‐bolus, Prophylactic (8 h sampling)	**Total plasma**: Obese: MIC 0.25–8 mg/L: 11.9–3.8 hours Non‐obese: MIC 0.25–8 mg/L: 10.2–3.5 h	S**CT** (both upper arms): Obese: MIC 0.25–8 mg/L: 11.0–1.4 hours Non‐obese: MIC 0.25–8 mg/L: 9.6–2.0 h	**Total plasma**, median: Obese: 54.0 mg/L Non‐obese: 63.9 mg/L	**SCT** (both upper arms), median: Obese: 12.6 mg/L Non‐obese: 18.6 mg/L	**SCT** (both upper arms), median: Obese: 0.35 Non‐obese: 0.48
Wittau 2015, Germany [47]	Meropenem	Obese patients after abdominal surgery (*n* = 2M3F), Age: 40, BMI: 54.2, S‐Cr: 73 umol/L	1 g every 8 h x 4, IV‐bolus, steady state (8 h sampling)			**Free plasma**: 24.6 mg/L	**SCT** (abdomen): 24.1 mg/L	
Abouelhassan 2023, USA [48]	Tebipenem	Healthy volunteers (*n* = 4M2F), Age: 34, BMI: 29, CrCl median: 129,5 mL/min, Patients with DFI (*n* = 5M1F), Age: 58, BMI: 31, CrCl median: 88 mL/min	600 mg x3, Oral therapy, Steady state (8 h sampling)			**Free plasma**: Healthy: 3.74 mg/LDFI: 3.40 mg/L	**SCT** (thigh): 2.85 mg/L**SCT** (DFI): 2.73 mg/L	**SCT** (thigh): 107.4 **SCT** (DFI): 90.0
Cephalosporin								
1st generation								
Dalley 2009, Australia [49]	Cephalothin	Healthy volunteers (*n* = 5), Age: 35	1 g, IV‐bolus, Prophylactic, (4 h sampling)		SCT (forearm): MIC 0.2 mg/L: 160 min	**Free plasma**: 17.6 mg/	**SCT** (forearm): 8.6 mg/L	
Mangum 2019, USA [37]	Cefazolin^1^	Rats, Sprague–Dawley (*n* = 9)	Cefazolin 35 mg/kg after TNK inflation and again after TNK release, IV‐bolus, Prophylactic and steady state (72 h sampling)			**Free plasma**: 155 ug/mL	**Skeletal muscle** (hind leg): non‐ischemic 51 ug/mL	**Skeletal muscle** (hind leg): non‐ischemic 0.29
Andreas 2015, Austria [50]	Cefazolin^2^	Patients undergoing CABG (*n* = 5M4F), Age: (67), BMI: 26.9, S‐Cr: 1.05 mg/dL	4 g + 2 g, IV‐bolus, Prophylactic (10 h sampling)			**Total plasma**: first dose: 273 ug/mL second dose: 135 ug/mL	**Cancellous bone** (left sternum): first dose: 112 ug/mL, second dose: 81 ug/mL **Cancellous bone** (right sternum): first dose: 159 ug/mL, second dose: 134 ug/mL	**Cancellous bone** (left sternum): 0.74 **Cancellous bone** (right sternum): 0.99
Brill 2014, The Netherlands [53]	Cefazolin	Obese: (*n* = 1M7F), Age 40.1, BMI: 47.0 Non‐obese: (*n* = 4M3F), Age: 53.7, BMI: 28.2	2 g, IV‐bolus, prophylactic (4 h sampling)					**SCT (**abdomen): Obese: 0.70 Non‐obese 1.02
Bates 2023, USA [51]	Cefazolin	Patients with open lower fractures (*n* = 7M1F) Age median: 32	2000 mg x4 (8‐h intervals), IV‐CI, Steady state (24 h sampling)		Injured lower limb: MIC 1 ug/mL: 100 % Control lower limb: MIC 1 ug/mL: 100%			
Bhalodi 2013, USA [52]	Cefazolin	Patients with lower limb infections q8h: (*n* = 5M1F), Age: 55, CrCl: 123.6 q8h: (*n* = 1 M), Age 50, CrCl 15.8	1 g every 8 h (*n* = 6), 2 g every 24 h (*n* = 1), IV‐bolus steady state (8 h sampling)			**Serum**: q8h: 94.52 ug/mL q24h: 196.83	**SCT** (wound)	**SCT** (wound): q8h: 1.2 q24h: 0.19
2nd generation								
Toma 2011, USA [54]	Cefoxitin	Obese patients undergoing abdominal or pelvic surgery (*n* = 2M12F), Age: 52, BMI: 43, Normal weight patients (*n* = 2F), Age: 44, BMI: 19 Healthy volunteers (*n* = 6M5F), Age: 25, BMI: 21	2 g for obese patients, 1 g for non‐obese, IV‐bolus, Prophylactic, (8 h sampling)			**Free plasma**: Obese: 258 ug/mL Healthy volunteers: 242 ug/mL All normal weight: 240 ug/mL	**SCT** (abdomen): Obese: 11 ug/ml Healthy volunteers: 20 ug/mL All normal weight: 22 ug/mL	**SCT** (abdomen): Obese: 0.08 ug/ml Healthy volunteers: 0.38 ug/mL All normal weight: 0.37 ug/mL
Hanberg 2016, Denmark [24]	Cefuroxime	Pigs (*n* = 10), Weight range: 75–77 kg	1500 mg, IV‐bolus, Prophylactic (8 h sampling)			**Free plasma**: 80.4 ug/mL	**Cancellous bone** (C3): 27.7 ug/mL **SCT** (thoracic wall): 34.4 ug/mL **Intervertebral disc** (C3‐C4): 11.9 ug/mL	**Cancellous bone** (C3): 0.78 **SCT** (thoracic wall): 0.94 **Intervertebral disc** (C3‐C4): 0.78
Hanberg 2020, Denmark [56]	Cefuroxime	Pigs (*n* = 16), Weight range: 73–77 kg	3 g cefuroxime given as one double dose (1X3 g) or two single doses (2X1.5 g) with a four‐hour interval, IV‐bolus, Prophylactic (8 h sampling)	**Free plasma**: Double dose x1: MIC 1–4 ug/mL: 71%–47% Single dose X2: MIC 1–4 ug/mL: 99%–72%	**Cancellous bone** (C3): Double dose x1: MIC 1–4 ug/mL: 80–59 %Single dose x2: MIC 1–4 ug/mL: 98%–79% **SCT** (neck): Double dose x1: MIC 1–4 ug/mL: 78%–51% Single dose x2: MIC 1–4 ug/mL: 100%–91% **Intervertebral disc** (C3‐C4): Double dose x1: MIC 1–4 ug/mL: 93%–67% Single dose x2: MIC 1–4 ug/mL: 97%–92%	**Free plasma**: Double dose x1: 179.8 ug/mLSingle dose x2: 81.1 ug/mL	**Cancellous bone** (C3): Double dose x1: 51.6 ug/mLSingle dose x2: 35.7 ug/mL **SCT** (neck) Double dose x1: 62.6 ug/mLSingle dose x2: 38.8 ug/mL **Intervertebral disc** (C3‐C4): Double dose x1: 27.6 ug/mLSingle dose x2: 14.9 ug/mL	**Cancellous bone** (C3): Double dose x1: 0.79Single dose x2: 0.73 **SCT** (neck): Double dose x1: 0.77Single dose x2: 0.87 **Intervertebral disc** (C3‐C4): Double dose x1: 0.61Single dose x2: 0.54
Hvistendahl 2022, Denmark [28]	Cefuroxime	Pigs (*n* = 8), Weight range: 74–77 kg	1.5 g, IV‐bolus, Prophylactic (8 h sampling)	**Free plasma**: MIC 4 mg/L: 1.9 h	**Cancellous bone** (Vertebral pedicle L1): MIC 4 mg/L: 1.6 h **Cannulated screw**: MIC 4 mg/L: 0 h	**Free plasma**: 59 ug/mL	**Cancellous bone** (Vertebral pedicle L1): 14 ug/mL **Cannulated screw**: 1 ug/mL	**Cancellous bone** (Vertebral pedicle L1): 48% **Cannulated screw**: 8%
Hvistendahl 2022, Denmark [60]	Cefuroxime	Pigs (*n* = 8), Weight range: 74–77 kg	1,5 mg, IV‐bolus, Prophylactic (8 h sampling)	**Free plasma**: MIC 1–4 ug/mL: 221–123 min	**Cancellous bone** (L5): Anterior column: MIC 1–4 ug/mL: 202‐97minPosterior column: MIC 1–4 ug/mL: 212‐93 min	**Free plasma**: 59 ug/mL	**Cancellous bone** (L5): Anterior column: 15 ug/mLPosterior column: 11 ug/mL	**Cancellous bone** (L5): Anterior column: 0.48Posterior column: 0.40
Kaspersen 2023, Denmark [62]	Cefuroxime	Pigs (*n* = 8), Weight range: 74–77 kg	1500 mg, IV‐bolus, Prophylactic (8 h sampling)	**Free plasma**: MIC 4 ug/mL: 123 min	**CSF**: MIC 4 ug/mL: 0 min **Epidural space**(L3‐L4): MIC 4 ug/mL: 115 min **Spinal cord** (L3‐L4): MIC 4 ug/mL: 58 min	**Free plasma**: 58 ug/mL	**CSF**: 1 ug/mL **Epidural space** (L3‐L4): 18 ug/mL **Spinal cord** (L3‐L4): 8ug/mL	**CSF**: 9% **Epidural space** (L3‐L4): 63% **Spinal cord** (L3‐L4): 32%
Hanberg 2020, Denmark [58]	Cefuroxime	Pigs (*n* = 6), Weight range: 78–82 kg	1500 mg, IV‐bolus, Prophylactic (6 h sampling)	**Free plasma**: MIC 4 ug/mL: 149 min	**Cancellous bone** (calcaneus): MIC 4 ug/mL: 213 min **SCT** (thigh): MIC 4 ug/mL: 183 min	**Free plasma**: 115.5 ug/mL	**Cancellous bone** (calcaneus) ISM: 38.5 ug/mLRDM: 39.5 ug/mL**SCT** (thigh) ISM: 68.3 ug/mLRDM: 68.8 ug/mL	**Cancellous bone** (calcaneus): ISM: 0.92RDM: 0.94**SCT** (thigh) ISM: 1.15 RDM: 1.16
Hanberg 2020, Denmark [59]	Cefuroxime	Pigs (*n* = 24), Weight range: 73–77 kg	1–5 g, IV‐bolus, Prophylactic (8 h sampling)	**Free plasma**: MIC 4 ug/mL: A: 145 min B: 147 min C: 142 min	**Cancellous bone** (calcaneus): MIC 4 ug/mL: A: non‐tourniquet: 187, tourniquet: 208 min B: non‐tourniquet: 213, tourniquet: 245 min C: non‐tourniquet: 206, tourniquet: 240 min **SCT** (Plantar): MIC 4 ug/mL: A: non‐tourniquet: 198, tourniquet: 198 min B: non‐tourniquet: 207, tourniquet: 204 min C: non‐tourniquet: 204, tourniquet: 226 min	**Free plasma**: A: 131 ug/mL B: 115 ug/mL C: 121 ug/mL	**Cancellous bone** (calcaneus): A: non‐tourniquet: 48 ug/mL, tourniquet: 32 ug/mL B: non‐tourniquet: 45 ug/mL, tourniquet: 45 ug/mL C: non‐tourniquet: 44 ug/mL, tourniquet: 67 ug/mL **SCT** (Plantar): A: non‐tourniquet: 55 ug/mL, tourniquet: 53 ug/mL B: non‐tourniquet: 58 ug/mL, tourniquet: 58 ug/mL C: non‐tourniquet: 59 ug/mL, tourniquet: 72 ug/mL	**Cancellous bone** (calcaneus): A: non‐tourniquet: 0.99, tourniquet: 0.98 B: non‐tourniquet: 1.20, tourniquet: 1.35 C: non‐tourniquet: 1.19, tourniquet: 1.56 **SCT** (Plantar): A: non‐tourniquet: 1.11, tourniquet: 1.06 B: non‐tourniquet: 1.17, tourniquet: 1.10 C: non‐tourniquet: 1.20, tourniquet: 1.50
Tøttrup 2014, Denmark [68]	Cefuroxime	Pigs (*n* = 15), Weight: 60 kg	1500 mg, IV‐bolus, Prophylactic (8 h sampling)			**Total plasma**: 154.4 ug/mL **Free plasma**: 109.9 ug/mL	**Cortical bone** (tibia): sealed drill holes: 4.8 ug/mLunsealed drill holes: 5.7 ug/mL **Cancellous bone** (femur): 22.9 ug/mL **SCT** (abdomen): 24.0 ug/mL	**Cancellous bone** (femur): 0.45 **SCT** (abdomen): 0.58
Jørgensen 2021, Denmark [61]	Cefuroxime	Pigs (*n* = 16), Weight range: 73–77 kg	Either one 15 min infusion of 3000 mg (Group 1) or two single 15 min infusions of 1500 mg administered 4 h apart (Group 2), IV‐bolus, Prophylactic (8 h sampling)	**Free plasma**: Double dose x1: MIC 4 ug/mL: 211 min, 44%Single dose x2: MIC 4 ug/mL: 324 min, 68%	**Cortical bone** (tibia): Double dose x1: MIC 4 ug/mL: 207 min, 43%Singe dose x2: MIC 4 ug/mL: 280 min, 58% **Cancellous bone** (tibia): Double dose x1: MIC 4 ug/mL: 253 min, 53%Singe dose x2: MIC 4 ug/mL: 394 min, 82%, **SCT** (thigh): Double dose x1: MIC 4 ug/mL: 237 min, 49%Singe dose x2: MIC 4 ug/mL: 383 min, 80%, **Synovial fluid** (knee): Double dose x1: MIC 4 ug/mL: 237 min, 49%Singe dose x2: MIC 4 ug/mL: 394 min, 82%	**Free plasma**, median: Double dose x1: 177.0 ug/mLSinge dose x2: 82.6 ug/mL	**Cortical bone** (tibia), median: Double dose x1: 11.0 ug/mLSinge dose x2: 7.9 ug/mL **Cancellous bone** (tibia), median: Double dose x1: 47.3 ug/mLSinge dose x2: 24.8 ug/mL **SCT** (thigh), median: Double dose x1: 74.8 ug/mLSinge dose x2: 34.5 ug/mL **Synovial fluid** (knee), median: Double dose x1: 62.2ug/mLSinge dose x2: 41.2 ug/mL	**Cortical bone** (tibia), median: Double dose x1: 0.28Singe dose x2: 0.26 **Cancellous bone** (tibia), median: Double dose x1: 0.67Singe dose x2: 0.63 **SCT** (thigh), median:Double dose x1: 0.91 Singe dose x2: 0.76 **Synovial fluid** (knee), median: Double dose x1: 0.81Singe dose x2: 0.80
Tøttrup 2015, Denmark [66]	Cefuroxime	Pigs (*n* = 12), Weight range: 72–79 kg	1500 mg, IV‐bolus (*n* = 6) or IV‐cont. (*n* = 6), Prophylactic (8 h sampling)	**Free plasma**: STI: MIC 0.5–4.0 ug/mL: 190–113 minCI: MIC 0.5–4.0 ug/mL: 465–465 min	**Cortical bone** (tibia): STI: MIC 0.5–4.0 ug/mL: 335–126 minCI: MIC 0.5–4.0 ug/mL: 457–0 min**Cancellous bone** (tibia): STI: MIC 0.5–4.0 ug/mL: 239–127 minCI: MIC 0.5–4.0 ug/mL: 463–61 min**SCT** (abdomen): STI: MIC 0.5–4.0 ug/mL: 249–142 minCI: MIC 0.5–4.0 ug/mL: 464–118 min	**Free plasma**: STI: 70.2 ug/mLCI: 51.4 ug/mL	**Cortical bone** (tibia): STI: 11.5 ug/mLCI: 2.5 ug/mL **Cancellous bone** (tibia): STI: 24.4 ug/mLCI: 6.1 ug/mL **SCT** (abdomen): STI: 45.4 ug/mLCI: 12.7 ug/mL	**Cortical bone** (tibia): STI: 0.45CI: 0.27 **Cancellous bone** (tibia): STI: 0.61CI: 0.38 **SCT** (abdomen): STI: 0.97CI: 0.53
Tøstesen 2022 Denmark [25]	Cefuroxime	Pigs (*n* = 18), Weight range: Group 1 (*n* = 6): 53–57 kgGroup 2 (*n* = 6): 73–77 kgGroup 1 (*n* = 6): 93–97 kg	20 mg/kg every 8 h, IB‐bolus, prophylactic (8 h sampling)	**Free plasma**: MIC 4 ug/mL Group 1: 137 min, 30% Group 2: 119 min, 26% Group 3: 116 min, 26%	**Cancellous bone** (scapula): MIC 4 ug/mL Group 1: 118 min, 26% Group 2: 154 min, 34% Group 3: 124 min, 28% **Skeletal muscle** (deltoideus): MIC 4 ug/mL Group 1: 146 min, 32% Group 2: 145 min, 32% Group 3: 109 min, 24% **SCT** (shoulder): MIC 4 ug/mL Group 1: 165 min, 37% Group 2: 110 min, 24% Group 3: 117 min, 26%	**Free plasma**: Group 1: 59 ug/mL Group 2: 52 ug/mL Group 3: 50 ug/mL	**Cancellous bone** (scapula): Group 1: 17 ug/ml Group 2: 18 ug/mL Group 3: 21 ug/mL **Skeletal muscle** (deltoideus): Group 1: 36 ug/mL Group 2: 39 ug/mL Group 3: 27 ug/mL **SCT** (shoulder): Group 1: 32 ug/mL Group 2: 26 ug/mL Group 3: 26 ug/mL	**Cancellous bone** (scapula): Group 1: 0.63 Group 2: 0.79 Group 3: 0.84 **Skeletal muscle** (deltoideus): Group 1: 0.95 Group 2: 1.26 Group 3: 1.27 **SCT** (shoulder): Group 1: 1.03 Group 2: 0.89 Group 3: 0.83
Tøstesen 2022, Denmark [65]	Cefuroxime	Pigs (*n* = 18), Weight range: Group 1 (*n* = 6): 53–57 kgGroup 2 (*n* = 6): 73–77 kgGroup 1 (*n* = 6): 93–97 kg	20 mg/kg every 8 h, IV‐bolus, Prophylactic (16 h sampling)	**Free plasma**, pooled comparison: 1. dosing interval: MIC 4 ug/mL: 1 24 min, 28%2. dosing interval: MIC 4 ug/mL: 160 min, 36%Total: MIC 4 ug/mL: 284 min, 32%	**Cancellous bone** (scapula), pooled comparison: 1. dosing interval: MIC 4 ug/mL: 130 min, 29%2. dosing interval: MIC 4 ug/mL: 205 min, 56%Total: MIC 4 ug/mL: 334 min, 37% **Dead space**: Group 11. dosing interval: MIC 4 ug/mL: 333 min2. dosing interval: MIC 4 ug/mL: 321 minGroup 21. dosing interval: MIC 4 ug/mL: 255 min2. dosing interval: MIC 4 ug/mL: 378 minGroup 31. dosing interval: MIC 4 ug/mL: 245 min2. dosing interval: MIC 4 ug/mL: 315 minpooled comparison: 1. dosing interval: MIC 4 ug/mL: 227 min, 62%2. dosing interval: MIC 4 ug/mL: 338 min, 75%Total: MIC 4 ug/mL: 605 min, 67%	**Free plasma**, pooled comparison: 1. dosing interval: 53 ug/mL2. dosing interval: 54 ug/mL	**Cancellous bone** (scapula), pooled comparison: 1. dosing interval: 18 ug/mL2. dosing interval: 18 ug/mL **Dead space**, pooled comparison: 1. dosing interval: 27 ug/mL2. dosing interval: 19 ug/mL	**Dead space** (shoulder): Group 11. dosing interval: 1.872. dosing interval:1 .13Group 21. dosing interval: 1.772. dosing interval: 1.70Group 31. dosing interval: 1.272. dosing interval: 1.19
Tøttrup 2016, Denmark [67]	Cefuroxime	Pigs with trauma‐induced implant‐associated S. aureus osteomyelitis (*n* = 10), Weight range: 67–77 kg	1500 mg, IV‐bolus, therapeutic (8 h sampling)			**Free plasma**: 63.2 mg/L	**Cancellous bone** (tibia): 22.9 mg/L **SCT** (leg): 30.7 mg/L **SCT** adjacent to infected implant: 30.5 mg/L **Cancellous bone** adjacent to infected bone cavity: 21.7 mg/L **Infected bone cavity**: 9.4 mg/L	**Cancellous bone** (tibia): 0.79 **SCT** (leg): 1.03 **SCT** adjacent to infected implant: 0.86 **Cancellous bone** adjacent to infected bone cavity: 0.74 **Infected bone cavity**: 0.50
Barbour 2009, USA [55]	Cefuroxime	Morbidly obese volunteers undergoing abdominal surgery (*n* = 6F), Age range: 19–76, BMI range: 44–53, S‐Cr range: 0.67–1.15 mg/dL	1500 mg, IV‐bolus, Prophylactic (6 h sampling)			**Free plasma**: 66.8 ug/mL	**Skeletal muscle** (thigh): 60.1 ug/mL **SCT** (thigh): 39.2 ug/mL	**Skeletal muscle** (thigh): 1.03 **SCT** (thigh):0.63
Skhirtladze‐Dworschak 2019, Austria [64]	Cefuroxime	Patients undergoing elective cardiac surgery. Bolus: (*n* = 2M4F), Age: 63, BMI: 26, S‐Cr: 0.9 mg/dL CI: (*n* = 3M3F), Age 69, BMI: 26, S‐Cr: 1.1 mg/dL	1.5 g 30 min before skin incision, after 12 h and 24 h, IV‐bolus, or 3 g IV‐CI after 1.5 g loading dose, prophylactic and steady state (12 h sampling)	**Free plasma,** median: MIC 2–16 mg/L Bolus: 360–60 min, 50–8% CI: 720–600 min, 100%–83%	**Skeletal muscle,** median (thigh): MIC 2–16 mg/L Bolus: 225–80 min, 31%–11% CI: 330–120 min, 46%–17% **SCT**, median (thigh): MIC 2–16 mg/L Bolus: 240–100 min, 33%–14% CI: 330–120 min, 46%–17%	**Total plasma,** median: Bolus: 77 mg/L CI: 90 mg/L **Free plasma**, median: Bolus: 58 mg/L CI: 68 mg/L	**Skeletal muscle,** median (thigh): Bolus: 26 mg/L CI: 32 mg/L **SCT**, median (thigh): Bolus: 21 mg/L CI: 33 mg/L	
Tøttrup 2019, Denmark [69]	Cefuroxime	Patients undergoing total knee replacement (*n* = 18), Bolus (*n* = 9 M): Age: 68.7, BMI: 30.6, P‐Cr: 76 umol/L CI(*n* = 9 M): Age: 70, BMI: 28.7, P‐Cr: 87 umol/L	1.5 g, Bolus (*n* = 9) or CI (*n* = 9) 0.5 g as loading dose over 5 min followed by CI of the remaining 1 g over 7 h and55 min), steady state (8 h sampling)					**Cortical bone** (tibia): STI: 0.35, CI: 0.65 **Cancellous bone** (tibia): STI: 1.03, CI: 1.15 **SCT** (thigh): STI: 0.52, CI: 0.69
Hanberg 2021, De [57]	Cefuroxime	Patients scheduled for hallux valgus or hallux rigidus surgery (*n* = 3M7F), BMI: 25, P‐Cr: 75 umol/L	Cefuroxime (1.5 g) IV‐bolus 15 min prior to tourniquet inflation, followed by a second dose 6 h later, Prophylactic (12 h sampling)	**Free plasma**: MIC 4 ug/mL: 318 min	**Cancellous bone** (calcaneus): Non‐tourniquet: MIC 4 ug/mL: 306 minTourniquet: MIC 4 ug/mL: 289 min **Skeletal muscle** (m. gastrocnemius): Non‐tourniquet: MIC 4 ug/mL: 320 minTourniquet: MIC 4 ug/mL: 316 min **SCT** (leg): Non‐tourniquet: MIC 4 ug/mL: 312 min Tourniquet: MIC 4 ug/mL: 322 min	**Free plasma**: 97 ug/mL	**Cancellous bone** (calcaneus): Non‐tourniquet: 59 ug/mL Tourniquet: 53 ug/mL **Skeletal muscle** (m. gastrocnemius): Non‐tourniquet: 61 ug/mL Tourniquet: 70 ug/mL **SCT** (leg): Non‐tourniquet: 58 ug/mL Tourniquet: 51 ug/mL	**Cancellous bone** (calcaneus): Non‐tourniquet: 0.84, Tourniquet: 0.88 **Skeletal muscle** (gastrocnemius): Non‐tourniquet: 0.92 Tourniquet: 0.98 **SCT** (leg): Non‐tourniquet: 1.1 Tourniquet: 0.96
Schwameis 2017, Au [63]	Cefuroxime	Patients undergoing elective knee arthroscopy (*n* = 8M2F), Age: 34.2, BMI: 26.0	1500 mg, IV‐bolus, Prophylactic (8 h sampling)	**Total plasma:** MIC 2–64 mg/L: 95.0%–5.0% **Free plasma:** MIC 2–64 mg/L: 85.4%–0%	**Skeletal muscle** (thigh): MIC 2–64 mg/L: 100–1.9 % **Synovial fluid** (knee): MIC 2–64 mg/L: 100%–0%	**Total plasma**: 103.0 mg/L **Free plasma**: 69.0 mg/L	**Skeletal muscle** (thigh): 67.1 mg/L	**Skeletal muscle** (thigh): 1.79 **Synovial fluid** (knee): 1.94
3rd generation								
Liu 2005, USA [71]	Cefixime	Healthy volunteers (*n* = 6 M)	400 mg, Oral therapy, Prophylactic (8 h sampling)			**Total plasma**: 3.4 mg/L	**Skeletal muscle** (thigh): 0.9 mg/L	**Skeletal muscle** (thigh): 0.84
Liu 2005, USA [70]	Cefpodoxime	Rats (*n* = 18) Weight range: 360–500 g	10 mg/kg, 20 mg/kg, IV‐bolus or 260 g/h IV‐cont. with loading dose of 6 mg/kg, Prophylactic, (5 h sampling)					**Skeletal muscle** (hind leg): 10 mg/kg: 67 20 mg/kg: 60 Cont: 0.70
Liu 2005, USA [71]	Cefdopoxime	Healthy volunteers (*n* = 6 M)	400 mg, Oral therapy, Prophylactic (8 h sampling)			**Total plasma**: 3.9 mg/L	**Skeletal muscle** (thigh): 2.4 mg/L	**Skeletal muscle** (thigh): 0.89
4th generation								
Steiner 2004, Austria [72]	Cefpirome	Healthy volunteers (*n* = 8 M), Age: 26.6, BMI: 22.3	2 g, IV‐Bolus, Prophylactic (3 h sampling)			**Free plasma**: Placebo: 188 mg/LNOR: 208 mg/L	**Skeletal muscle** (thigh): Placebo: 131 mg/L, NOR: 139 mg/L **SCT** (thigh): Placebo: 116 mg/L, NOR: 118 mg/L	**Skeletal muscle** (thigh): Placebo: 0.82 mg/L, NOR: 0.83 mg/L **SCT** (thigh): Placebo:0.81 mg/L, NOR: 0.76 mg/L
5th generation								
Matzneller 2016, Austria [73]	Ceftaroline	Healthy volunteers (*n* = 12 M), Age range: 24–50, BMI: q8 h 23.2, q12 h 22.2	600 mg x2 or x3 IV‐Bolus, steady state (8 or 12 h sampling) CPT‐F was administered every 8 h as a 2‐h infusion in the first group (intensified dosing regimen, further referred to here as the q8 h regimen) and every 12 h as a 1‐h infusion in the second group		**Skeletal muscle** (thigh): MIC 1 mg/L Single administration q12h: 48.3%, q8h: 60.75 %Repeated administrationq12h: 56.8%, q8h: 75.6% **SCT** (thigh): MIC 1 mg/L Single administrationq12h: 47.4%, q8h: 64.6 %Repeated administrationq12h: 54.3%, q8h: 75.1%	**Total plasma**: Single administrationq12h: 22.6 mg/Lq8h: 15.3 mg/LRepeated administrationq12h: 22.0 mg/Lq8h: 14.7 mg/L	**Skeletal muscle** (thigh): Single administrationq12h: 6.1 mg/L, q8h: 5.5 mg/LRepeated administrationq12h: 7.1 mg/L, q8h: 6.3 mg/L **SCT** (thigh): Single administrationq12h: 6.3 mg/L, q8h: 4.8 mg/LRepeated administrationq12h: 8.5 mg/L, q8h: 6.8 mg/L	**Skeletal muscle** (thigh): Single administrationq12h: 0.50, q8h: 0.49 Repeated administrationq12h: 0.66, q8h: 0.67 **SCT** (thigh): Single administrationq12h: 0.53, q8h: 0.47 Repeated administrationq12h: 0.75, q8h: 0.75
Barbour 2009, USA [74]	Ceftobiprole	Healthy volunteers (*n* = 6F9M), Age range: 20–34	500 mg, IV‐Bolus, Prophylactic (24 h sampling)		**Skeletal muscle** (thigh): MIC 2 mg/L: >50% **SCT** (thigh): MIC 2 mg/L > 50%	**Total plasma**: 25.8 mg/L **Free plasma**: 20.2 mg/L	**Skeletal muscle** (thigh): 14.0 mg/L **SCT** (thigh): 9.61 mg/L	**Skeletal muscle** (thigh): 0.69 **SCT** (thigh): 0.49
Al Jalali 2021, Austria [75]	Ceftolozane/Tazobactam	Healthy volunteers (*n* = 8 M), Age: 30.6, BMI: 25.0, GFR, Cockcroft‐Gault: 126.1 mL/min	1000 mg ceftolozane, 500 mg tazobactam every 8 h, IV‐bolusl, Steady state and prophylactic (8 h sampling)	**Free plasma**: Ceftolozane Single doseMIC 0.5–32 mg/L: 100–16%Multiple doseMIC 0.5–32 mg/L: 100–17 %Tazobactam Single doseMIC 4 mg/L: 21%Multiple dose MIC 4 mg/L: 21%	**Skeletal muscle** (thigh): Ceftolozane Single dose: MIC 0.5–32 mg/L: 100–13% Multiple dose: MIC 0.5–32 mg/L: 100%–14% Tazobactam Single dose: MIC 4 mg/L: 22% Multiple dose: MIC 4 mg/L: 22% **SCT** (thigh): Ceftolozane Single dose: MIC 0.5–32 mg/L: 10–15% Multiple dose: MIC 0.5–32 mg/L: 100–15% Tazobactam Single dose: MIC 4 mg/L: 21% Multiple dose: MIC 4 mg/L: 21%	**Total plasma**: Ceftolozane Single dose: 57.5 mg/L Multiple dose: 58.2 mg/L Tazobactam Single dose: 16.2 mg/L Multiple dose: 14.8 mg/L	**Skeletal muscle** (thigh): Ceftolozane Single dose: 30.7 mg/L Multiple dose: 37.6 mg/L Tazobactam Single dose: 5.8 mg/L Multiple dose: 8.0 mg/L **SCT** (thigh): Ceftolozane Single dose: 22.5 mg/LMultiple dose: 40.2 mg/L Tazobactam Single dose: 7.5 mg/L Multiple dose: 8.4 mg/L	**Skeletal muscle** (thigh): Ceftolozane Single dose: 0.74 Multiple dose: 0.92 Tazobactam Single dose: 0.67 Multiple dose: 0.89 **SCT** (thigh): Ceftolozane Single dose: 0.90 Multiple dose: 0.88 Tazobactam Single dose: 0.81 Multiple dose: 0.87
Monogue 2017, USA [76]	Ceftolozane/Tazobactam	Patients with DFI (*n* = 1F9M), Age median: 53, BMI median: 33.4, CrCl median: 108.4 mL/min Healthy volunteers (*n* = 2F4M), Age median: 28, BMI median: 24.0, CrCl median: 121.5 mL/min	1.5 g every 8 h, IV‐bolus, Steady state (8 h sampling)		**SCT** (DFI), median: MIC 2–4 ug/mL: 100–99.8% **SCT** (thigh), median: MIC 2–4 ug/mL: 100%–93.8%	**Total plasma**, median: Ceftolozane DFI: 52.2 ug/mL Healthy: 91.5 ug/mL Tazobactam DFI: 14.2 ug/mL Healthy: 17.5 ug/mL	Ceftolozane **SCT** (DFI), median: 33.8 ug/mL **SCT** (thigh), median: 39.2 ug/mL Tazobactam **SCT** (DFI): 7.1 ug/mL **SCT** (thigh): 6.3 ug/mL	Cetolozane: **SCT** (DFI), median: 0.75 **SCT** (thigh), median: 0.88 Tazobactam: **SCT** (DFI), median: 1.18 **SCT** (thigh), median: 0.85
Penicillin								
Rasmussen 2024, Denmark [77]	Penicillin V Penicillin G	Pigs (*n* = 12), Weight range: 68–75 kg	0,8 g every 6 h oral or 1,2 g every 6 h IV‐bolus Prophylactic, steady state (18 h sampling)		**Cancellous bone** (tibia): MIC: 0.125–0.5 mg/L Oral 1. dose: 40%–7% IV 1. dose: 97%–81% Oral 3. dose: 42%–18% IV 3. dose: 100%–84% **SCT** (leg): MIC: 0.125–0.5 mg/L Oral 1. dose: 72%–31% IV 1. dose: 97–75% Oral 3. dose: 44%–26% IV 3. dose: 100%–89%			
Marchand 2005, France [78]	Amoxicillin	Rats (*n* = 11), Weigh: 333 g	50 mg/kg, IV bolus, prophylactic (3 h sampling)					**Skeletal muscle** (hind leg): 0,80
Jonsson 2014, Germany [79]	Cloxacillin	Patients with CLI (*n* = 4M4F), Age: 78 Healthy volunteers (1M2F), Age: 67	4 g, IV‐bolus, Prophylactic, (4 h sampling)			**Total serum**, median: Healthy: 397 ug/mL CLI: 305.5 ug/mL	**SCT** (medial malleol), median: Healthy: 20 ug/mL CLI: 24 ug/mL **Skeletal muscle** (gastrocnemius), median: Healthy: 30 ug/mL CLI: 49 ug/mL **SCT** (pectoral region), median: Healthy: 29 ug/mL CLI: 32 ug/mL	**SCT** (medial malleol): Healthy: 0.11 CLI: 0.11 **Skeletal muscle** (gastrocnemius): Healthy: 0.12 CLI: 0.16 **SCT** (pectoral region): Healthy: 0.13 CLI: 0.16
Hansen 2018, Denmark [80]	Dicloxacillin	Healthy volunteers (*n* = 6 M), Age range: 25–27, BMI range: 20–28	2 g, IV‐bolus, Prophylactic (6 h sampling)	**Free plasma**, median: MIC 0.125 ug/mL: 3.2 h	**Skeletal muscle** (lateral vastus), median: MIC 0.125 ug/mL: 4.1 h **SCT** (abdomen), median: MIC 0.125 ug/mL: 3.2 h			
Bendtsen 2021, Denmark [23]	Flucloxacillin	Pigs (*n* = 16), Weight range: 78–82 kg	1 g every 6 h, IV bolus or Oral, Prophylactic and steady state (24 h sampling)	**Free plasma**: MIC 0,5–2 ug/mL: IV 1. dose: 77–44 min, 23%–13% Oral 1. dose: 1–0 min, 0.3%–0% IV 4. dose:100–49 min, 30%–15% Oral 4. dose: 11–0 min, 3%–0%	**Cortical bone** (tibia): MIC 0,5–2 ug/mL: IV 1. dose: 61–0.5 min, 18%–0.2% Oral 1. dose: 26–0 min, 8%–0% IV 4. dose: 7–0 min, 2–0% Oral 4. dose: 0–0 min, 0–0% **Cancellous bone** (tibia): MIC 0,5–2 ug/mL: IV 1. dose: 106–0 min, 32–0% Oral 1. dose: 10–0 min, 3%–0% IV 4. dose: 126–9 min, 38%–3% Oral 4. dose: 0–0 min, 0%–0% **Synovial fluid** (knee): MIC 0,5–2 ug/mL: IV 1. dose: 114–39 min, 35–12% Oral 1. dose: 0–0, 0%–0% min IV 4. dose: 76–33 min, 23%–10% Oral 4. dose: 0–0 min, 0%–0% **SCT** (leg): MIC 0,5–2 ug/mL: IV 1. dose: 149–16 min, 45%–5% Oral 1. dose: 0–0 min, 0%–0% IV 4. dose: 108–15 min, 33%–5% Oral 4. dose: 0–0 min, 0%–0%	**Free plasma:** IV 1. dose: 6.4 ug/mL Oral 1. dose: 0.3 ug/mL IV 4. dose: 7 ug/mL Oral 4. dose: 0.5 ug/mL	**Cortical bone** (tibia): IV 1. dose: 0.7 ug/mL Oral 1. dose: 0.3 ug/mL IV 4. dose: 0.3 ug/mL Oral 4. dose: 0.1 ug/mL **Cancellous bone** (tibia): IV 1. dose: 1.3 ug/mL Oral 1. dose: 0.2 ug/mL IV 4. dose: 1.6 ug/mL Oral 4. dose: 0.3 ug/mL **Synovial fluid** (knee): IV 1. dose: 2.5 ug/mL Oral 1. dose: 0.2 ug/mL IV 4. dose: 2.6 ug/mL Oral 4. dose: 0.2 ug/mL **SCT** (leg): IV 1. dose: 1.9 ug/mL Oral 1. dose: 0.2 ug/mL IV 4. dose: 1.9 ug/mL Oral 4. dose: 0.2 ug/mL	**Cortical bone** (tibia): IV 1. dose: 0.5 Oral 1. dose: 1.7 IV 4. dose: 0.2 Oral 4. dose: 0.6 **Cancellous bone** (tibia): IV 1. dose: 0.6 Oral 1. dose: 1 IV 4. dose: 0.6 Oral 4. dose: 0.4 **Synovial fluid** (knee): IV 1. dose: 0.9 Oral 1. dose: 0.9 IV 4. dose: 0.6 Oral 4. dose: 0.7 **SCT** (leg): IV 1. dose: 0.9 Oral 1. dose: 0.8 IV 4. dose: 0.6 Oral 4. dose: 0.6
Bendtsen 2022, Denmark [81]	Flucloxacillin	Pigs (*n* = 16), Weight range: 78–82 kg	1 g every 6 h for 24 h, IV‐bolus or oral, steady state (8 h sampling)	**Free plasma**: MIC 0.125–2 ug/mL: IV: 205–49 min 62%–15% Oral: 108–0 min 33%–0%	**Cancellous vertebral bone** (C3): MIC 0.125–2 ug/mL: IV: 227–0 min, 69%–0% Oral: 41–0 min, 12–0% **Intervertebral disc** (C3‐C4): MIC 0.125–2 ug/mL: IV: 155–0 min, 47%–0% Oral: 4–0 min, 1–0% **SCT** (neck): MIC 0.125–2 ug/mL: IV: 200–27 min, 61%–8% Oral: 38–0 min, 12–0%	**Free plasma**: IV: 7.0 ug/mL Oral: 0.5 ug/mL **Total plasma**: IV: 15.7 ug/mL Oral: 1.5 ug/mL	**Cancellous vertebral bone** (C3): IV: 0.9 ug/ml Oral: 0.1 ug/mL **Intervertebral disc** (C3‐C4): IV: 0.6 ug/mL Oral 0.06 ug/mL **SCT** (neck): IV: 2.2 ug/mL Oral: 0.15 ug/mL	**Cancellous vertebral bone** (C3): IV: 0.43 Oral: 0.38 **Intervertebral disc** (C3‐C4): IV: 0.32 Oral 0.33 **SCT** (neck): IV: 0.74 Oral: 0.53
Knudsen 2021, Denmark [83]	Piperacillin	Pigs (*n* = 8), Weight range: 78–82 kg	4/0.5 g piperacillin/tazobactam, IV bolus, prophylactic (8 h sampling)	**Free plasma**: MIC 4–16 ug/mL: 382–232 min 85%–52%	**Cancellous bone** (tibia): MIC 4–16 ug/mL ISM: 398–255 min 88%–57% RDM: 408–254 91%–56% min **SCT** (leg): MIC 4–16 ug/mL ISM: 423–295 min, 94%–66% RDM: 421–283 min, 94%–63%	**Free plasma**: 362 ug/mL	**Cancellous bone** (tibia): ISM: 123 ug/mL RDM: 136 ug/mL **SCT** (leg): ISM: 124 ug/mL RDM: 115 ug/mL	**Cancellous bone** (tibia): ISM: 0.86 RDM: 0.90 **SCT** (leg): ISM: 0.98 RDM: 0.92
Lilleøre 2023, Denmark [84]	Piperacillin	Pigs (*n* = 6), Weight range: 86–90 kg	4/0.5 g piperacillin/tazobactam every 6 h, IV‐bolus, steady state (6 h sampling)	**Total plasma**: MIC 8–16 ug/mL: 238–162 min, 72%–49%	**Cancellous bone** adjacent to screw (tibia) MIC 8–16 ug/mL: 243–177 min, 74%–54% Contralateral **cancellous bone** (tibia) MIC 8–16 ug/mL: 178–112 min, 54%–34% **Cannulated screw**: MIC 8–16 ug/mL: 180–53 min, 55%–16%	**Total plasma**, median: 571 ug/mL	**Cancellous bone** adjacent to screw (tibia), median: 49 ug/mL Contralateral **cancellous bone** (tibia), median: 45 ug/mL **Cannulated screw**, median: 11 ug/mL	**Cancellous bone** adjacent to screw (tibia), median: 0.35 Contralateral **cancellous bone** (tibia), median: 0.31 **Cannulated screw**, median: 0.16
Petersen 2022, Denmark [85]	Piperacillin	Pigs (*n* = 16), Weight range: 86–90 kg	piperacillin/tazobactam IV bolus (4/0.5 g every 6 h) or CI (4/0.5 g as a bolus followed by 12/1.5 g) for 18 h steady state (6 h sampling)	**Free plasma**: MIC 4–16 ug/mL: Bolus: 300–163 min 91%–49% Cont: 328–316 min 99%–96%	**Cancellous bone** (C3): Bolus: MIC 4–16 ug/mL: 237–82 min, 72%–25% Cont: MIC 4–16 ug/mL: 327–92 min, 99%–28% **Skeletal muscle** (C2‐C4): Bolus: MIC 4–16 ug/mL: 307–151 min, 93%–46% Cont: MIC 4–16 ug/mL: 328–294 min, 99%–89% **SCT** (neck): Bolus: MIC 4–16 ug/mL: 325–197 min, 98%–60% Cont: MIC 4–16 ug/mL: 328–310 min, 99%–94% **Intervertebral disc** (C3‐C4): Bolus: MIC 4–16 ug/mL: 307–80 min, 93%–24% Cont: MIC 4–16 ug/mL: 326–123 min, 99%–37%	**Total plasma**: Bolus: 681.0 ug/mL Cont: 117.6 ug/mL	**Cancellous bone** (C3): Bolus: 40.5 ug/mL Cont: 23.5 ug/mL **Skeletal muscle** (C2‐C4): Bolus: 126.3 ug/mL Cont: 33.3 ug/ml **SCT** (neck): Bolus: 101.8 ug/mL Cont: 31.6 ug/mL **Intervertebral disc**: Bolus: 22.1 ug/mL Cont: 20.4 ug/mL	**Cancellous bone** (C3): Bolus: 0.21 Cont: 0.35 **Skeletal muscle** (C2‐C4): Bolus: 0.57 Cont: 0.72 **SCT** (neck): Bolus: 0.60 Cont:0.74 **Intervertebral disc**: Bolus: 0.24 Cont: 0.37
Rasmussen 2023, Denmark [86]	Piperacillin	Pigs (*n* = 16), Weight range: 86–90 kg	4/0.5 g piperacillin/tazobactam every 6 h (IV bolus, *n* = 8) or a continuous infusion (*n* = 8) of 12 g/1.5 g during 7.5 h after initial bolus (4/0,5 g), steady state (6 h sampling)	**Free plasma**: cont: MIC 4–64 mg/L: 330–69 min 100%–21% bolus: MIC 4–64 mg/L: 300–69 min 91%–21%	**Cortical bone** (tibia), median: cont: MIC 4–64 mg/L: 65–0 min, 20%–0% bolus: MIC 4–64 mg/L: 133–0 min 40%–0%, **Cancellous bone** (tibia): cont: MIC 4–64 mg/L: 330–0 min, 100%–0% bolus: MIC 4–64 mg/L: 244–7 min, 74%–2%, **Synovial fluid** (knee), median: cont: MIC 4–64 mg/L: 330–0 min, 100%–0% bolus: MIC 4–64 mg/L: 272–30 min, 82%–9% **SCT** (hind leg), median: cont: MIC 4–63 mg/L: 330–10 min, 100%–3% bolus: MIC 4–64 mg/L: 253–30 min, 77%–9%	**Free plasma**, median: cont: 72 mg/L bolus: 499 mg/L	**Cortical bone** (tibia), median: cont: 3 mg/L bolus: 5 mg/L **Cancellous bone** (tibia), median: cont: 22 mg/L bolus: 46 mg/L **Synovial fluid** (knee), median: cont: 35 mg/L bolus: 88 mg/L **SCT** (hind leg), median: cont: 40 mg/L bolus: 77 mg/L	**Cortical bone** (tibia), median: cont: 0.04 bolus: 0.07 **Cancellous bone** (tibia), median: cont: 0.41 bolus: 0.33 **Synovial fluid** (knee), median: cont: 0.45 bolus: 0.38 **SCT** (hind leg), median: cont: 0.56 bolus: 0.34
Legat 2005, Austria [82]	Piperacillin	Patients suffering from DFI (*n* = 4F2M), Age: 72.3	4/0.5 g piperacillin/tazobactam, IV‐bolus every 8 h, steady state (8 h sampling)			**Total plasma**: Piperacillin: 341.0 mg/L Tazobactam: 15.8 mg/L	**SCT** (thigh) Piperacillin: 91.7 mg/L Tazobactam: 5.2 mg/L **SCT** (DFI) Piperacillin: 102.2 mg/L Tazobactam: 10.4 mg/L	
Matzneller 2020, Austria [87]	Temocillin	Healthy volunteers (*n* = 8 M), Age 32.9, BMI: 24,4	2 g, IV‐bolus, prophylactic (12 h sampling)	**Free plasma**: MIC 8–16 mg/L: 18.4%–7.6%	**SCT** (thigh): MIC 8–16 mg/L: 74.5%–34.5% **Skeletal muscle** (thigh): MIC 8–16 mg/L: 41.2%–13.9%	**Total plasma**: 233.5 mg/L **Free plasma**: 34.6 mg/L	**SCT** (thigh): 34.2 mg/L **Skeletal muscle** (thigh): 20.6 mg/L	
Cyclic lipopeptide								
Kim 2008, USA [88]	Daptomycin	Healthy volunteers w.(*n* = 4F2M, Age: 53, BMI: 28, CrCl: 75.8) or w/o diabetes (*n* = 4F2M, Age: 40, BMI: 26.4, CrCl: 96.2)	4 mg/kg, IV‐bolus, prophylactiv(24 h sampling)			**Total plasma**: Diabetic: 67.8 ug/mL Nondiabetic: 62.4 ug/mL	**SCT** (thigh): Diabetic: 4.3 ug/mL Nondiabetic: 3.8 ug/mL	**SCT** (thigh): Diabetic: 0.93 Nondiabetic: 0.74
Traunmüller 2010, Austria [89]	Daptomycin	Patients with DFI (*n* = 3F7M), morbidly obese Age: 53, BMI: 45.3, S‐Cr: 1.1 mg/dL Normal, Age: 61.7, BMI: 30.1, S‐Cr: 1.1 mg/dL	6 mg/kg daily for 4 days, IV‐bolus, steady state (16 h sampling)			**Total Plasma**: 72.9 ug/mL **Free plasma**: 6.3 ug/mL	**Cancellous bone** (metatarsal): 4.7 mg/L **SCT** (leg): healthy: 4.1 mg/L inflamed: 4.0 mg/L	**Cancellous bone** (metatarsal): 1.17 **SCT** (leg): healthy: 1.54, inflamed: 1.06
Fluoroquinolone								
Joukhadar 2005, Austria [90]	Ciprofloxacin	Healthy volunteers (*n* = 12 M), Age: 29.5	200 mg IV‐bolus, prophylactic (5 h sampling)			**Total plasma**: 2.49 mg/L	**SCT** (thigh): warmed: 1.97 mg/L normal temp: 1.06 mg/L	
Zhao2019, China [91]	Gemifloxacin	Rats, Healthy (*n* = 6), Infected (*n* = 6), weight: 200 g	18 mg/kg, IV‐bolus, prophylactic (9 h sampling)	**Free plasma**: Healthy: MIC 0.03 mg/L: 100% Infected: MIC 0.03 mg/L: 100%	**Skeletal muscle** (thigh): Healthy MIC 0.03 mg/L: 100% Infected: MIC 0.03 mg/L: 100%	**Free plasma**: Healthy: 5027.43 ng/mL Infected: 4726.20 ng/mL	**Skeletal muscle** (thigh) Healthy: 1766.37 ng/mL Infected: 1675.19 ng/mL	**Skeletal muscle** (thigh) Healthy: 0.54 Infected: 0.52
Islinger 2004, Austria [92]	Gemifloxacin	Healthy volunteers (*n* = 12 M), Age: 29	320 mg, oral, Prophylactic (10 h sampling interval)			**Total plasma**: 1.2 mg/L **Free plasma**: 0.5 mg/L	**Skeletal muscle** (thigh): 0.5 mg/L **SCT** (thigh): 8.0 mg/L	**Skeletal muscle** (thigh): 1.7 **SCT** (thigh): 2.4
Bellmann 2004, Austria [93]	Levofloxacin	Patients with soft tissue infections (*n* = 6M2F), Age: 56, pCr: 1.12 mg/dL	500 mg, iV‐bolus, Prophylactic (10 h sampling interval)			**Total plasma**: 8.37 ug/mL	**SCT**: inflamed: 5.45 ug/mL healthy (thigh): 4.42 ug/mL	
Mangum 2019, USA [37]	Moxifloxacin	Rats (*n* = 9)	15 mg/kg, IV‐bolus, prophylactic and steady state (4 h sampling)			**Free plasma**: 63 ug/mL	**Skeletal muscle** (hind leg) non‐ischemic limb: 10 ug/mL	**Skeletal muscle** (hind leg) non‐ischemic limb: 0.17
Slater 2022, Denmark [29]	Moxifloxacin	Pigs, (*n* = 20), weight range: 78–82 kg	mono therapy (*n* = 10): 400 mg daily for 3 days combination therapy (*n* = 10): 450 mg rifampicin x 2 daily for 7 days co‐administered 400 mg of moxifloxacin daily for 3 days. of, oral therapy, steady state (24 h sampling)			**Free plasma**, median: Monotherapy: 0.92 ug/mL Combination therapy: 0.77 ug/mL	**Cortical bone** (tibia), median: Monotherapy: 0.26 ug/mL Combination therapy: 0.04 ug/mL **Cancellous bone** (tibia), median: Monotherapy: 0.43 ug/mL Combination therapy: 0.16 ug/mL **SCT** (leg), median: Monotherapy: 0.51 ug/mL Combination therapy: 0.12 ug/mL **Synovial fluid** (knee), median: Monotherapy: 0.65 ug/mL Combination therapy: 0.27ug/mL	**Cortical bone** (tibia), median: Monotherapy: 0.17 Combination therapy: 0.03 **Cancellous bone** (tibia), median: Monotherapy: 0.37 Combination therapy: 0.15 **SCT** (leg), median: Monotherapy: 0.59 Combination therapy: 0.12 **Synovial fluid** (knee), median: Monotherapy: 0.59 Combination therapy: 0.24
Slater 2020, Denmark [94]	Moxifloxacin	Pigs, (*n* = 20), weight range: 78–82 kg	mono therapy (*n* = 10): 400 mg daily for 3 days combination therapy (*n* = 10): 450 mg rifampicin x 2 daily for 7 days co‐administered 400 mg of moxifloxacin daily for 3 days. of, oral therapy, steady state (24 h sampling)			**Free plasma**: Monotherapy: 0.92 mg/L Combination therapy: 0.77 mg/L	**Cancellous bone** (C3): Monotherapy: 0.79 mg/L Combination therapy: 0.17 mg/L **Intervertebral disc** (C3‐4): Monotherapy: 0.51 mg/L Combination therapy: 0.13 mg/L **SCT** (foreleg): Monotherapy: 0.91 mg/L Combination therapy: 0.31 mg/L	**Cancellous bone** (C3): Monotherapy: 0.69 mg/L Combination therapy: 0.21 mg/L **Intervertebral disc** (C3‐4): Monotherapy: 0.41 mg/L Combination therapy: 0.16 mg/L **SCT** (foreleg): Monotherapy: 0.84 mg/L Combination therapy: 0.37 mg/L
Vittrup 2024, Denmark [95]	Moxifloxacin	Pigs (*n* = 16), Implant‐associated osteomyelitis, Weight range: 61–69 kg	Group RM: 7 days of intravenous antibiotic treatment of either rifampicin 450 mg twice daily combined with moxifloxacin 400 mg once daily Group M: moxifloxacin 400 mg once daily, IVbolus, steady state (8 h sampling)			**Free plasma**: M: 1.7 ug/mL RM: 2.9 ug/mL **Total plasma**: M: 5.2 ug/mL RM: 4.8 ug/mL	**Implant cavity** (tibia): M: 1.3 ug/mL RM: 1.0 ug/mL **Infected cancellous bone** (tibia): M: 1.2 ug/mL RM: 0.8 ug/mL **Noninfected cancellous bone** (tibia): M: 0.9 ug/mL RM: 0.7 ug/mL **Infected SCT** (leg): M: 2.2 ug/mL RM: 1.8 ug/mL **Noninfected SCT** (leg): M: 2.1 ug/mL RM:1.8 ug/mL	**Implant cavity** (tibia): M: 1.6 RM: 1.3 **Infected cancellous bone** (tibia): M: 1.5 RM: 1.1 **Noninfected cancellous bone** (tibia): M: 1.2 RM: 1.0 **Infected SCT** (leg): M: 2.0 RM: 1.5 **Noninfected SCT** (leg): M: 1.7 RM: 1.3
Glycylcycline								
Dorn 2022, Germany [96]	Tigecycline	Obese (*n* = 15), 5M10F, Age, median: 46, BMI, median: class II(*N* = 1):35.5 class III (*n* = 14): 52.4, eGFR, median: 92.4, S‐Cr, median: 0.86 mg/dL Non‐obese (*n* = 14, 5M9F, Age, median: 44, BMI, median: 23.8, eGFR: 102, S‐Cr: 0.74 mg/dL)	100 mg, IV‐bolus, prophylactic (8 h sampling interval)			**Total plasma**: Obese L: 1.31 mg/L Non‐obese: 2.27 mg/L **Free plasma**: Obese: 0.768 mg/L Non‐obese: 0.888 mg/L	**SCT** (both upper arms): Obese: 0.135 mg/L Non‐obese: 0.204 mg/L	**SCT** (both upper arms): Obese: 0.375 Non‐obese: 0.628
Bulik 2010, USA [97]	Tigecycline	Patients with mild chronic diabetic foot infections (*n* = 6M2F), Age: 63, BMI: 30.4	100 mg loading dose, 50 mg every 12 h, IV‐bolus, steady state (12 h sampling)			**Total plasma**: 0.42 ug/mL **Free plasma**: 0.16 ug/mL	**SCT** (thigh): 0.18 ug/mL **SCT** (DFI): 0.16 ug/mL	**SCT** (thigh): 99.94% **SCT** (DFI): 100%
Glycopeptides								
Häfeli 2011, Canada [98]	Vancomycin	Rabbits, (*n* = 4), Weight range: 2.2–3.5 kg	20 mg/kg, IV‐bolus, Prophylactic (8 h sampling)			**Total plasma**: 72.1 ug/mL	**SCT** (upper back): 32.1 ug/mL	
Bue 2015, Denmark [26]	Vancomycin	Pigs, (*n* = 8), p‐Cr:115–136 umol/L, 65–75 kg	1000 mg, IV‐bolus, Prophylactic (12 h sampling)			**Free plasma**: 34.2 ug/mL	**Cortical bone** (tibia): 9.4 ug/mL **Cancellous bone** (tibia): 17.2 ug/mL **SCT** (leg): 27.2 ug/mL	**Cortical bone** (tibia): 0.36 **Cancellous bone** (tibia): 0.65 **SCT** (leg): 1.01
Bue 2018, Denmark [99]	Vancomycin	Pigs, (*n* = 8), 78–82 kg	1000 mg over 100 min, IV‐bolus, Prophylactic (8 h sampling)			**Free plasma**: 40.0 ug/mL	**Cancellous bone** (C3): 12.4 ug/mL **Intervertebral disc** (C3‐C4): 6.6 ug/mL **SCT** (thigh): 18.0 ug/mL	**Cancellous bone** (C3): 0.46 **Intervertebral disc** (C3‐C4): 0.24 **SCT** (thigh): 0.60
Slater 2023, Denmark [43]	Vancomycin^3^	Pigs, (*n* = 8), Weight range: 78–82 kg	1000 mg over 100 min, IV‐bolus, Prophylactic (8 h sampling)	**Free plasma** MIC 1–4 ug/mL: 449–446 min, 100%–99%	**Cancellous bone** (C3): MIC 1–4 ug/mL: 388–214 min, 86%–47% **Intervertebral disc** (C3‐C4) MIC 1–4 ug/mL: 311–45 min, 69%–10% **Paravertebral skeletal muscle**: MIC 1–4 ug/mL: 433–306 min, 96%–68% **SCT** (neck): MIC 1–4 ug/mL: 435–365 min, 97%–81%	**Free plasma**: 35 ug/mL	**Cancellous bone** (C3): 6 ug/mL **Intervertebral disc** (C3‐C4): 3 ug/mL **Paravertebral skeletal muscle**: 11 ug/mL **SCT** (neck): 17 ug/mL	**Cancellous bone** (C3): 0.24 **Intervertebral disc** (C3‐C4): 0.12 **Paravertebral skeletal muscle**: 0.36 **SCT** (neck): 0.53
Vittrup 2022, Denmark [44]	Vancomycin^3^	Pigs (*n* = 8), Weight range: 78–82 kg	1000 mg over 100 min, IV‐bolus, Prophylactic (8 h sampling)	**Free plasma** MIC 1–4 ug/mL: 449–446 min, 100%–99%	**Cortical bone** (tibia) MIC 1–4 ug/mL: 208–30 min, 46%–7% **Cancellous bone** (tibia): MIC 1–4 ug/mL: 403–296 min, 89%–66% **SCT** (Tibia): MIC 1–4 ug/mL: 441–416 min, 98%–93%	**Free plasma**: 35 ug/mL	**Cortical bone** (tibia): 7 ug/mL **Cancellous bone** (tibia): 14 ug/mL **SCT** (Tibia): 22 ug/mL	
Vittrup 2022, Denmark [45]	Vancomycin^3^	Pigs (*n* = 8), Weight range: 78–82 kg	1000 mg over 100 min, IV‐bolus, Prophylactic (8 h sampling)	**Free plasma** MIC 1–4 ug/mL: 449–446 min, 100%–99%	**Cannulated screw** (tibia) MIC 1–4 ug/mL: 88–25 min, 19%–6% **Cancellous bone adjacent to screw** (tibia): MIC 1–4 ug/mL: 372–3 min, 83%–1% **Cancellous bone on contralateral leg** (Tibia): MIC 1–4 ug/mL: 403–296 min, 62%–32%	**Free plasma**: 35 ug/mL	**Cannulated screw** (tibia): 1 ug/mL **Cancellous bone adjacent to screw** (tibia): 3 ug/mL **Cancellous bone on contralateral leg** (Tibia): 14 ug/mL	
Bue 2018, Denmark [27]	Vancomycin	Pigs, traumatically induced S. aureus osteomyelitis (*n* = 8), 75–86 kg	1000 mg, IV‐bolus, therapeutic (8 h sampling)			**Free plasma**, median: 43.2 ug/mL	**Cancellous bone** (tibia), median: 15.7 ug/mL **Cancellous bone** adjacent to infection (tibia), median: 9.9 ug/mL **Infected bone cavity** (tibia), median: 4.1ug/mL **SCT** (leg), median: 25.4 ug/mL. **SCT** adjacent to infection (leg), median: 22.0 ug/mL	**Cancellous bone** (tibia), median: 0.59 **Cancellous bone** adjacent to infection (tibia), median: 0.42 **Infected bone cavity** (tibia), median: 0.20 **SCT** (leg), median: 0.87 **SCT** adjacent to infection (leg), median: 0.74
Bue 2018, Denmark [101]	Vancomycin	Patients undergoing total knee replacement, (*n* = 10 M), p‐Cr: 82 umol/L	1000 mg, IV‐bolus, prophylactic (8 h sampling)			**Free plasma**, median: 34.4 ug/mL	**Cortical bone** (tibia), median: 4.0 ug/mL **Cancellous bone** (tibia), median: 10.8 ug/mL **SCT** (thigh), median: 6.6 ug/mL	**Cortical bone** (tibia), median: 0.17 **Cancellous bone** (tibia), median: 0.45ug/mL **SCT** (thigh), median: 0.31
Housman 2015, USA [100]	Vancomycin	Patient with lower limb infections, (*n* = 7M2F), Age: 54, BMI: 30, CrCl: 101.4 mL/min	Loading dose: 25 mg/kg, individual dose to target a trough concentration of 10–20 ug/mL. Mean dose: 12,8 mg/kg, IV‐bolus, Steady state (12 h sampling)			**Serum:** 8 h dosing (*n* = 1): 23.36 ug/mL 12 h dosing (*n* = 7): 35.7 ug/mL 24 h dosing (*n* = 1): 33.76 ug/mL	**SCT** (wound): 8 h dosing: 13.5 ug/mL 12 h dosing: 17.4 (6.0) ug/mL 24 h dosing: 11.1 ug/mL	
Lipoglycopeptide								
Matzneller 2016, Austria [102]	Telavancin	Healthy volunteers (*n* = 8 M), Age: 27.6, BMI: 22.2	10 mg/kg, IV‐bolus, Prophylactic (24 h sampling)			**Total plasma**: 73.6 mg/L **Free plasma**: 13.8 mg/L	**Skeletal muscle** (thigh): 4.3 mg/L **SCT** (thigh): 3.8 mg/L	**Skeletal muscle** (thigh): 0.93 **SCT** (thigh): 0.51
Macrolide								
Matzneller 2013, Austria [103]	Azithromycin	Healthy volunteers (*n* = 6 M), Age: 29.0, BMI: 22.83	500 g x1 for 3 days, Oral, Prophylactic and steady state (8 h sampling			**Total plasma:** Day 1: 460.68 ng/mL Day 3: 565.53 ng/mL Day 5: 27.80 ng/mL Day 10: 8.15 ng/mL	**Skeletal muscle** (thigh): Day 1: 30.21 ng/mL Day 3: 39.74 ng/mL Day 5: 19.18 ng/mL Day 10: 10.70 ng/mL **SCT** (thigh): Day 1: 22.50 ng/mL Day 3: 23.91 ng/mL Day 5: 6.76 ng/mL Day 10: 5.60 ng/mL	
Traunmüller 2007, Austria [104]	Clarithromycin	Healthy volunteers (*n* = 6 M), Age: 25–37	single dose: 250 mg, multiple dose: 500 mg x 2 daily for 3–5 days, Prophylactic and steady state (8 h sampling)			**Total plasma**: Single dose: 1.09 mg/mL Multiple doses: 2.21 mg/mL **Free plasma**: Single dose: 0.31 mg/mL Multiple doses: 0.58 mg/mL	**Skeletal muscle** (thigh): Single dose: 0.08 mg/mL Multiple doses: 0.23 mg/mL **SCT** (thigh): Single dose: 0.07 mg/mL Multiple doses: 0.23 mg/mL	**Skeletal muscle** (thigh): Single dose: 0.42 Multiple doses: 0.41 **SCT** (thigh): Single dose: 0.29 Multiple doses: 0.39
Krasniqi 2012, Austria [105]	Erythromycin	Healthy volunteers (*n* = 6 M), Age: 29.17, BMI: 22.57	500 mg x 4 daily for 3 days, oral, prophylactic, steady state and after end therapy (10 h sampling)			**Free plasma**: Day 1 (prophylactic): 1371 ng/mL Day 3 (steady state): 2544 ng/mL Day 5 (after end therapy): 34.2 ng/mL	**Skeletal muscle** (thigh): Day 1 (prophylactic): 42.9 ng/mL Day 3 (steady state): 154 ng/mL Day 5 (after end therapy): 3.58 ng/mL **SCT** (thigh): Day 1 (prophylactic): 55.5 ng/mL Day 3 (steady state): 209 ng/mL Day 5 (after end therapy): 5.11 ng/mL	
Gattringer 2004, Austria [106]	Telithromycin	Healthy volunteers, (*n* = 10 M), Age 18–40	800 mg, Oral, Prophylactic (8 h sampling)			**Total plasma**: 1.2 mg/L **Free plasma**: 0.1 mg/L	**Skeletal muscle** (thigh): 0.2 mg/L **SCT** (leg): 0.2 mg/L	**Skeletal muscle** (thigh): 1.5 **SCT** (leg): 2.1
Traunmüller 2009, Austria [107]	Telithromycin	Healthy volunteers, (*n* = 10 M), Age 18–41	800 mg for 5 days, Oral, Steady state (8 h sampling)			**Total plasma**, median: 1.73 mg/L **Free plasma**, median: 0.52 mg/L	**Skeletal muscle** (thigh), median: 0.13 mg/L **SCT** (thigh), median: 0.19 mg/L	**Skeletal muscle** (thigh), median: 0.27 **SCT** (thigh): 0.58
Oxalidinone								
Stolle 2008, Denmark [112]	Linezolid	Pigs (*n* = 10), 38–43 kg	600 mg, IV‐bolus, prophylactic (6 h sampling)			**Total plasma**: 28.1 ug/mL	**Cancellous bone** (tibia), median: right: 13.2 ug/mL, left: 15.5 ug/mL	**Cancellous bone** (tibia), median: right: 0.63, left: 0.6
Dehghanyar 2005, Austria [113]	Linezolid	Healthy volunteers (*n* = 5F5M), Age: 55	600 mg x2 daily, for 3–5 days, fist dose IV, the rest oral, prophylactic and steady state (8 h sampling)			**Total plasma**: Single dose: 14.1 mg/L Multiple doses: 19.5 mg/L **Free plasma**: Single dose: 12.8 mg/L Multiple doses: 17.1 mg/L	**Skeletal muscle** (thigh): Single dose: 13.5 mg/L Multiple doses: 15.4 mg/L **SCT** (thigh): Single dose: 18.1 mg/L Multiple doses: 12.9 mg/L	**Skeletal muscle** (thigh): Single dose: 1.3 Multiple doses: 1.0 **SCT** (thigh): Single dose: 1.4 Multiple doses: 0.9
Islinger 2005, Austria [114]	Linezolid	Healthy volunteers (*n* = 4F5M), Age: 56	1200 mg a day for 3–5 days, oral therapy, steady state (8 h sampling)			**Free plasma:** Fasting: 17.5 mg/L Non‐fasting: 15.5 mg/L	**Skeletal muscle** (thigh): Fasting: 12.6 mg/L Non‐fasting: 15.0 mg/L, **SCT** (thigh): Fasting: 12.2 mg/L Non‐fasting: 11.8 mg/L	**Skeletal muscle** (thigh): Fasting: 0.9 Non‐fasting: 1.0 **SCT** (thigh): Fasting: 0.9 Non‐fasting: 0.9
Andreas 2015, Austria [50]	Linezolid	Patients undergoing CABG (*n* = 4F5M), Age: 67, BMI: 26.9, P‐Cr: 1.05 mg/dL	600 mg linezolid over 30 min, starting 60 min prior to skin incision. Second dose 12 h after, IV‐bolus, prophylactic (12 h sampling)			**Total plasma**: first dose: 22.1 ug/mL second dose: 17.4 ug/mL	**Cancellous bone** (left sternum): first dose: 10.9 ug/mL second dose: 13.4 ug/mL, **Cancellous bone** (right sternum): first dose: 12.6 ug/mL second dose: 14.0 ug/mL	**Cancellous bone** (left sternum): 0.82 **Cancellous bone** (right sternum): 1.02
Schwameis 2017, Austria [115]	Linezolid	Patients undergoing elective knee arthroscopy (*n* = 2F8M), Age: 34.2, BMI: 26.0	600 mg, IV‐bolus, prophylactic (8 h sampling)			**Total plasma**: 17.6 mg/L **Free plasma**: 15.5 mg/L	**Skeletal muscle** (thigh): 12.3 mg/L **Synovial fluid** (knee): 8.0 mg/L	**Skeletal muscle** (thigh): 0.98 **Synovial fluid** (knee): 0.76
Eslam 2014, Austria [108]	Linezolid	T2DM Patients with DFI (*n* = 3F7M), Age: 59–81, BMI 32.7	600 mg every 12 h for 3 days, IV‐bolus, first dose and steady state (8 h sampling)			**Total plasma**: Single dose: 16.4 mg/L Multiple doses: 27.4 mg/L	**SCT** (thigh) Single dose: 6.7 mg/L Multiple doses: 14.9 mg/L **SCT** (DFI): Single dose: 6.6 mg/L Multiple doses: 12.7 mg/L	
Wiskirchen 2011, USA [109]	Linezolid	Diabetic patients with chronic lower limb infections (*n* = 9 M), Age: 55.1, BMI: 31.8, CrCl: <30 mL/min	600 mg every 12 h for 2 days, IV‐bolus, steady state (12 h sampling)			**Free plasma**: 11.99 ug/mL	**SCT** (wound): 13.45 ug/mL **SCT** (thigh): 14.4 ug/mL	
Traunmüller 2010, Austria [111]	Linezolid	Patients with type II diabetes presenting with deep‐seated bacterial foot infections (*n* = 3 M), Age: 60–67, BMI 26.3–37.2	600 mg x 2 daily for 3 days, IV‐bolus, steady state (12 h sampling)			**Total plasma**, median: 22.4 mg/L, **Free plasma**, median: 17.8 mg/L	**Cancellous bone** (metatarsal), median: 17.0 mg/L **SCT** (Healthy), median: 13.9 mg/L **SCT** (Inflamed), median: 17.4 mg/L	**Cancellous bone** (metatarsal), median: 1.09, **SCT** (healthy), median: 1.32 **SCT** (inflamed), median: 1.12
Koomanachai 2011, USA [110]	Linezolid	Diabetic patients with lower‐extremity ulcers (*n* = 1F1M), Age: 62–63, BMI: 36.5–41.5, CrCl: 30–34.9 mL/min	600 mg before and after HBO2 course, Oral, therapeutic (12 h sampling)					**SCT** (inflamed): Patient 1 pre‐HBO2:0.474, Post‐HBO2:0.950 Patient 2 pre‐HBO2: 0.0479, Post‐HBO2: 0.757
Sahre 2012, USA [116]	Tedizolid	Healthy volunteers (*n* = 7F5M), Age: 24, BMI: 20–29	600 mg, Oral, Prophylactic (12 h sampling)			**Total plasma**: 5.4 mg/L **Free plasma**: 0.69 mg/L	**Skeletal muscle** (thigh): 0.74 mg/L **SCT** (thigh): 0.66 mg/L	**Skeletal muscle** (thigh): 1.2 **SCT** (thigh): 1.1
Stainton 2018, USA [117]	Tedizolid	Diabetic patients with DFI (*n* = 5F5M), Age: 51, BMI: 31.5, ClCr: 90 mL/min Healthy volunteers (*n* = 3F3M), Age: 33, BMI: 28.5, ClCr: 102 mL/min	200 mg every 24 h for 3 days, oral, steady state (24 h sampling)			**Total plasma**: DFI: 1.5 mg/L, Healthy: 2.7 mg/L		**SCT** (DFI), median: 1.1 **SCT** (thigh), median: 0.8
Phosphonic antibiotics								
Dorn 2019, Germany [118]	Fosfomycin	Obese patients undergoing bariatric surgery (*n* = 5M8F), Age median: 48, BMI median: 42.0, eGFR: 101.3 Non‐obese patients undergoing abdominal surgery (*n* = 5M9F), Age median: 52, BMI median: 26.2, eGFR: 95.9	8 g, IV‐bolus, Prophylactic (8 h sampling)	**Free plasma**: MIC 32 mg/L: Obese: 8.3h^4^ Non‐obese: 9.4h^4^	**SCT** (upper arm): MIC 32 mg/L: Obese: 9.9h^4^ Non‐obese: 11.1h^4^	**Free plasma**: Obese: 468 mg/L Non‐obese: 594 mg/L	**SCT** (upper arm): Obese: 235 mg/L Non‐obese: 511 mg/L	**SCT** (upper arm): Obese: 0.86 Non‐obese: 1.27
Schintler 2009, Austria [119]	Fosfomycin	T2DM patients scheduled for partial bone resection due to bacterial foot infection and osteomyelitis (*n* = 3F6M), Age:48–83, BMI: 22.4–37.1	100 mg/kg, IV‐bolus, Therapeutic (6 h sampling)		**Cancellous bone** (metatarsal): MIC 4–32 mg/L: 117%–50%	**Free plasma**: 377.3 mg/L	**Cancellous bone** (metatarsal): 96.4 mg/L **SCT** (leg): 185.1 mg/L	**Cancellous bone** (metatarsal): 0.43 **SCT** (leg): 0.76
Pleuromutilin								
Zeitlinger 2016, Austria [120]	Lefamulin	Healthy volunteers (*n* = 12 M), Age: 26.9, BMI: 23.0	150 mg, IV‐bolus, prophylactic (24 h sampling)			**Total plasma,** median: 2539 ng/mL **Free plasma**, median: 330.1 ng/mL	**Skeletal muscle** (thigh), median: 138.1 ng/mL **SCT** (thigh), median: 145.1 ng/mL	
Polypeptide antibiotics								
Matzneller 2015, Austria [121]	Colistin	Healthy volunteers (*n* = 6), Age: 27.0, BMI: 21.8	2.5 million IU, IV‐bolus, prophylactic (8 h sampling)			**Total plasma**: 1.4 ug/mL Free plasma: 0.06 ug/mL	**Skeletal muscle** (thigh): 0.1 ug/mL **SCT** (thigh): 0.04 ug/mL	
Tetracycline								
Gill 2022, USA [122]	Omadacycline	Diabetic patients with DFI (*n* = 3F5M), Age: 55, BMI: 30 Healthy volunteers (*n* = 6, 2F4M), Age:45, BMI: 36	day 1 loading dose of 200 mg IV‐bolus infusion, on dag 2 and 3, 300 mg oral, steady state (24 h)			**Free plasma**: DFI: 0.57 mg/L, Healthy: 1.14 mg/L		**SCT** (DFI): 0.66 **SCT** (thigh): 0.54

Animals are healthy unless otherwise stated. All data are presented as means unless otherwise stated, standard deviation and confidence intervals can be found in original articles. The colours are to differentiate between groups, antibiotics of the same groups will be presented in the same colour.

CI, Continuous Infusion; ISM, Internal Standard Method; RDM, Retrodialysis by Drug Method; STI, Short‐Term Infusion.

^1^ Administered as a combination of cefazolin, moxifloxacin and ertapenem.

^2^ Administered with linezolid.

^3^ Administered as a combination of meropenem and vancomycin.

^4^ UC/MIC calculated for a daily dose of 24 g of fosfomycin in plasma or subcutaneous tissue of obese and non‐obese surgical patients.

#### Aminoglycosides

##### Amikacin

An adequate C_max_ in plasma, synovial fluid and SCT was described in a prophylactic setting in horses, using ultrafiltration [33].

##### Gentamicin

Comparable cancellous bone concentrations to those of bone specimens were described in pigs in a prophylactic setting. Also, similar SCT concentrations to those previously described in humans were observed [34].

#### Beta‐lactams

Beta‐lactams are the most widely used class of antibiotics and the most investigated.

##### Carbapenems

###### Doripenem

Sufficient T >MIC in muscle and SCT in healthy volunteers mimicking a prophylactic situation have been reported [35].

###### Ertapenem

Three studies have assessed the distribution of ertapenem [36, 37, 38]. In rats, the use of a tourniquet resulted in reduced distribution during reperfusion [37]. In healthy volunteers, acceptable T >MIC in muscle and SCT was described, when mimicking a prophylactic situation, [36]. In steady state, inflamed SCT in diabetic foot patients presented with higher concentrations compared to healthy SCT [38].

###### Imipenem

High penetration to muscle was found in rats in both prophylactic (hydrated/non‐hydrated and hypovolaemic/normovolaemic states) and infected settings [39, 40, 41].

###### Meropenem

Four studies investigated meropenem concentrations in healthy pigs [42, 43, 44, 45]. In a prophylactic situation, short T >MIC was observed in tibial cancellous bone, cortical bone, the internal deadspace of a cannulated screw, vertebral cancellous bone, SCT, paravertebral muscle and the intervertebral disc when co‐administered with vancomycin [42, 43, 44, 45].

In obese patients (BMI >35 kg/m^2^), weight was not found to have an effect on the prophylactic T >MIC in SCT after elective abdominal surgery [46]. However, in steady state and for morbidly obese patients (BMI >40 kg/m^2^), SCT tissue penetration was found to vary greatly [47].

###### Tebipenem

At steady state, high tissue penetration to SCT of both healthy and diabetic patients with ongoing foot infections has been described [48].

#### Cephalosporins

##### First generation

###### Cephalothin

Incomplete tissue penetration was reported to SCT of healthy volunteers in a prophylactic setting [49].

###### Cefazolin

Five studies have assessed the distribution of cefazolin [37, 50, 51, 52, 53]. In rats, the exposure was found to decline with reperfusion following the use of a tourniquet [37].

High penetration was reported in the sternal cancellous bone of patients undergoing coronary artery bypass grafting [50]. In patients undergoing laparoscopic abdominal surgery, drug penetration was impaired in SCT of obese patients [53].

100% T >MIC in injured and control legs were described for patients with open lower limb fractures for at least 24 h after the first postoperative dose [51]. In patients with active lower limb infections, high penetration to the wound margin has been described at steady state [52].

##### Second generation

###### Cefoxitin

In patients undergoing abdominal or pelvic surgery, prophylactic SCT concentrations and tissue penetration were negatively affected by weight [54].

###### Cefuroxime

Eighteen studies have assessed the distribution of cefuroxime [24, 25, 28, 55, 56, 57, 58, 59, 60, 61, 62, 63, 64, 65, 66, 67, 68, 69]. Twelve of these were conducted in healthy pigs simulating prophylactic settings [24, 25, 28, 56, 58, 59, 60, 61, 62, 65, 66, 68], while one study was performed in an infected porcine setting [67].

Penetration to C3 vertebral cancellous bone, SCT and intervertebral disc was delayed and incomplete in two studies [24, 56]. Two single bolus doses (2 × 1.5 g with a 4 h interval) displayed higher T >MIC in an 8 h dosing interval when compared with one double dose (1 × 3 g) [56].

Subtherapeutic prophylactic concentrations inside a cannulated pedicle screw were found in lumbar vertebral cancellous bone and a cannulated pedicle screw, with higher concentrations in the opposite non‐instrumented vertebral pedicle, but incomplete tissue penetration remained [28, 60]. At the same lumbar level, significantly lower concentrations and shorter T >MIC were observed in cerebrospinal fluid, spinal cord and epidural space [62].

Delayed penetration, prolonged half‐life and longer T >MIC in calcaneal cancellous bone compared to SCT have been described when comparing two methods of microdialysis calibration [58] and timing of tourniquet inflation [59]. Cefuroxime administration 15–45 min prior to tourniquet inflation resulted in sufficient T >MIC in calcaneal cancellous bone and SCT throughout a 90‐min tourniquet application [59].

A heterogeneous distribution has been described when comparing SCT, tibial cortical and cancellous bone [68]. Two single bolus doses (2 × 1.5 g with a 4 h interval) also provided longer T >MIC in synovial fluid, SCT, tibial cortical and cancellous bone when compared to one double dose (1 × 3 g) [61]. Continuous infusion provided superior T >MIC in SCT, tibial cortical and cancellous bone in comparison with short‐term infusion when administered in an equivalent dosage (1.5 g) [66]. Weight‐based dosing provided comparable T >MIC in cancellous bone of the scapula, muscle and SCT [25]. In an induced orthopaedic dead space, T >MIC and elimination were longer compared to cancellous bone of the scapula and plasma [65].

One study was performed in an implant‐associated osteomyelitis porcine model, describing a decrease in penetration along with the progression of infection [67].

Five studies have been performed in clinical settings [55, 57, 63, 64, 69]. High penetration to muscle and SCT of morbidly obese volunteers undergoing abdominal surgery and adequate perioperative concentration have been shown [55]. In patients undergoing elective cardiac surgery, continuous infusion yielded a longer time above MIC in SCT and muscle compared to bolus infusion [64]. This has also been demonstrated in SCT, cortical and cancellous bone of patients undergoing elective total knee replacement surgery [69]. In patients scheduled for hallux valgus or rigidus surgery, when administered 15 min prior to tourniquet inflation, no effect of tourniquet application on SCT, muscle or calcaneal cancellous bone concentrations was observed [57].

In the setting of elective knee arthroscopy, T >MIC in synovial fluid and muscle tissue exceeded that of plasma [63].

##### Third generation

###### Cefpodoxime and cefixime

In rats, impaired muscle penetration of cefpodoxime was described after both bolus and continuous infusion [70]. In healthy volunteers, higher muscle penetration of cefpodoxime compared to cefixime has been reported [71].

##### Fourth generation

###### Cefpirome

Intravenous noradrenaline administration had no effect on muscle and SCT distribution of healthy volunteers [72].

##### Fifth generation

###### Ceftaroline

In healthy volunteers at steady state, dosing at 8 h intervals provided longer T >MIC and higher drug exposure in muscle and SCT compared to administration at 12 h intervals [73].

###### Ceftobiprole

High penetration was reported in muscle and SCT of healthy volunteers [74].

###### Ceftolozane

Two studies assessed ceftolozane concentrations in healthy volunteers when co‐administered with tazobactam [75, 76]. The studies mimicked prophylactic and steady‐state settings, exhibiting high penetration to muscle and SCT in both situations. In diabetic foot infection, patient tissue inflammation did not affect SCT penetration [76].

#### Penicillins

##### Penicillin V and G

In a porcine model, short T >MIC was observed for penicillin V (oral administration) in cancellous bone and SCT, while penicillin G (intravenous administration) provided longer T >MIC in both compartments in both the first and third dosing intervals [77].

##### Amoxicillin

In rats, high muscle penetration was reported in a prophylactic setting [78].

##### Cloxacillin

Comparable concentrations were described in muscle and SCT of the lower extremities in patients suffering from critical limb ischemia and healthy controls [79].

##### Dicloxacillin

Sufficient T >MIC was described in muscle and SCT of healthy volunteers [80].

##### Flucloxacillin

In comparison with oral administration, intravenous administration provided longer but still short T >MIC in both prophylactic and steady‐state settings in porcine muscle, tibial cancellous bone, synovial fluid, SCT, intervertebral disc and vertebral cancellous bone [23, 81].

##### Piperacillin

Five studies assessed piperacillin concentrations when co‐administered with tazobactam [82, 83, 84, 85, 86]. Four were performed in healthy pigs [83, 84, 85, 86], describing high tissue penetration, longer steady state T >MIC in cancellous bone, synovial fluid, muscle, SCT, intervertebral disc and plasma when administered as a continuous infusion in comparison with bolus infusion and low concentrations in the internal dead space of a cannulated screw [83, 84, 85, 86].

In patients with diabetic foot infections, a high SCT penetration has been reported in a steady‐state setting [82].

##### Temocillin

Sufficient T >MIC was described in muscle and SCT of healthy volunteers in a prophylactic setting [87].

#### Cyclic lipopeptides

##### Daptomycin

Healthy volunteers and diabetic patients presented with comparable SCT tissue concentrations and penetration in a prophylactic setting [88]. In steady state, high penetration to metatarsal cancellous bone and SCT in patients with diabetic foot infection was described regardless of inflammation status [89].

#### Fluoroquinolones

##### Ciprofloxacin

For healthy volunteers, higher concentrations have been found in heated SCT compared to normal temperature SCT [90].

##### Gemifloxacin

Reduced muscle concentrations have been described with no difference between healthy and MRSA‐infected rats [91]. In healthy volunteers, high muscle and SCT penetration of was reported [92].

##### Levofloxacin

No differences in healthy and inflamed SCT concentrations were found in acute soft tissue infections without ischemic or necrotic regions in a perioperative surgical setting [93].

##### Moxifloxacin

Four studies evaluated the distribution of moxifloxacin in animal models [29, 37, 94, 95]. In rats, reduced distribution following the use of a tourniquet was found [37]. In healthy tissues in a pig model, co‐administration with rifampicin reduced the steady‐state concentrations in synovial fluid of the knee, cancellous, and cortical tibial bone, vertebral cancellous bone, intervertebral disc and SCT in comparison with administration of moxifloxacin alone [29, 94]. This effect of rifampicin‐induced reduction in tissue concentrations was less pronounced when investigated in an implant‐associated osteomyelitis porcine model [95].

#### Glycylcyclines

##### Tigecycline

In a surgical prophylactic setting, obese patients presented with lower SCT concentrations and tissue penetration than non‐obese patients [96]. In steady state of mild chronic diabetic foot infections, equal penetration was described in inflamed and healthy SCT [97].

#### Glycopeptides

##### Vancomycin

Nine available studies have examined vancomycin [26, 27, 43, 44, 45, 98, 99, 100, 101]. The ultrafiltration method was used to examine prophylactic SCT exposure in rabbits describing similar concentrations in SCT and plasma [98].

Five studies were performed in a prophylactic setting on healthy pigs [26, 43, 44, 45, 99]. Delayed and incomplete tissue penetration was found in the tibial cortical and cancellous bone, vertebral cancellous bone, SCT and intervertebral disc, with the intervertebral disc and cortical bone displaying the lowest values [26, 99]. The same findings were reproduced when co‐administered with meropenem [43, 44]. The presence of a cannulated screw in cancellous bone reduced the exposure in the adjacent cancellous bone [45].

In a porcine model of implant‐associated osteomyelitis, a significant reduction in vancomycin penetration was observed following the progression of the infection [27].

Two clinical studies have been conducted [100, 101]. In patients undergoing elective total knee replacement surgery, penetration was delayed and incomplete in SCT, cortical and cancellous bone [101]. In infected SCT of the lower limb, the concentrations were found to be lower than those of plasma [100].

#### Lipoglycopeptides

##### Telavancin

During an entire dosing interval, satisfactory concentrations in SCT and muscle tissue of healthy volunteers have been observed [102].

#### Macrolide

Five studies assessed the concentrations of macrolides in SCT and muscle of healthy volunteers [103, 104, 105].

##### Azithromycin

In both a prophylactic setting and at steady state, subtherapeutic concentrations in muscle and SCT after oral administration were observed [103].

##### Clarithromycin

Lower concentrations and penetration in soft tissues than those of plasma were described in both a prophylactic setting and at steady state [104].

##### Erythromycin

Steady state provided higher peak concentrations in muscle and SCT than a prophylaxis dose, and a rapid decrease in concentrations after the end of treatment was observed [105].

##### Telithromycin

In SCT and muscle of healthy volunteers, high concentrations and penetration were reported after a prophylaxis dose [106], while lower SCT and muscle concentrations were reported at steady state [107].

#### Oxazolidinones

##### Linezolid

Nine studies have assessed linezolid [50, 108, 109, 110, 111, 112, 113, 114, 115]. Low cancellous bone penetration was found in a prophylactic setting in a porcine model [112].

High penetration to SCT and muscle of healthy volunteers was reported in both a prophylactic setting (intravenous administration) and at steady state (oral administration) [113, 114]. Insufficient concentrations have been described in the sternal cancellous bone of patients undergoing cardiothoracic surgery [50]. In elective knee arthroscopy patients, muscle and synovial fluid were reported with high penetration [115].

No differences were found in healthy and inflamed SCT of diabetic patients [108], and SCT concentrations equaled those of plasma in steady state but with substantial inter‐patient variability [108, 109]. High steady‐state concentrations were also presented in the metatarsal bone of infected diabetic foot patients [111]. Applying hyperbaric treatment to diabetic patients improved penetration to both healthy and inflamed SCT [110].

##### Tedizolid

Healthy volunteers were described as having high SCT and muscle penetration in a prophylactic setting [116]. No difference in SCT penetration between healthy volunteers and diabetic patients has been found [117].

#### Phosphonic antibiotics

##### Fosfomycin

In abdominal surgery, obese patients presented with lower SCT concentrations than non‐obese patients in a prophylactic setting [118]. Sufficient SCT and metatarsal cancellous bone concentrations and penetration were described in diabetic patients undergoing partial toe amputations [119].

#### Pleuromutilins

##### Lefamulin

High SCT and muscle penetration in healthy volunteers have been found after a prophylactic dose [120].

#### Polypeptides

##### Colistin

Healthy volunteers have reported low prophylactic concentrations in SCT, muscle and plasma [121].

#### Tetracyclines

##### Omadacycline

No differences were found at steady state between SCT of healthy volunteers and inflamed tissue of infected diabetic foot patients [122].

## DISCUSSION

In orthopaedics, efficacious antibiotic treatment is determined by the clinical setting, the invading bacteria, the surgical intervention, the status of the host, the exposed tissues and drug‐specific PK/PD abilities [123]. This review summarizes the current literature on antibiotic distribution in orthopedically relevant tissues and settings using dynamic sampling methods. We included 97 studies (43 different antibiotic drugs) encompassing both small and large animal models (42%) and clinical contexts, including healthy and infected tissues (21%) and prophylactic and steady‐state situations (35%). Microdialysis emerged as the predominant sampling method in 98% of the studies. The majority of the presented antibiotics (80%) were only assessed once or twice. Among the most extensively studied antibiotics were cefuroxime (18 studies, 19% of included studied), linezolid (9 studies, 9% of included studied) and vancomycin (9 studies, 9% of included studied).

### Tissue compartments

The cortical bone and the intervertebral disc were among the reported compartments with the lowest antibiotic exposure, while SCT and muscle demonstrated the best antibiotic exposure. Given the different tissue composition, density and vascularity, this may not be surprising but illustrates the justification for not generalizing the antibiotic treatment: no one‐size‐fits‐all model exists that can encompass all orthopedically relevant settings. When it comes to the insertion of orthopaedic implants, this concept may be further challenged, as exemplified by the few studies that found an effect of screw insertion in the adjacent cancellous bone antibiotic distribution. Future studies investigating different implants in different tissues and under different conditions are warranted.

### Way of administration

It was not unexpected that intravenous administration facilitated higher target tissue concentrations and a higher probability of reaching defined PK/PD targets than oral administration. Oral administration is dependent on absorption and first‐pass metabolism, which may explain these findings. Continuous infusion and dosing according to weight demonstrated to improve intravenous administration (i.e. antibiotic target tissue exposure) and appear as simple, inexpensive and easy tools to apply.

### PK/PD target

The sufficiency of the reported antibiotic tissue concentrations and PK/PD‐data is inherently tied to the applied target. In most clinical scenarios, the optimal target is not known, as the target discussions often remain approximations based on in vitro studies, animal studies, PK/PD‐modelling and case reports. The main reason for this is that the host–bacterial interaction is extremely complex to understand and difficult to generalize to broad populations. Within this also often lies an underappreciation of the importance of individually approaching the bacterial species. For example, the distribution of planktonic MIC values varies significantly within the specific bacterial species and will thus vary between patients infected with the same bacteria. Furthermore, MIC values are obtained under standardized in vitro conditions and do not readily reflect that of in vivo.

In all antibiotic PK/PD‐data discussions, the measure of tissue penetration seems to add limited value. This is especially true if the concentrations are based on single tissue specimens, as these can theoretically be taken at any time along the concentration‐time curve. Dynamic assessment embraces most of these limitations, although an adequate tissue distribution (penetration) does not imply whether the relevant PK/PD targets are reached or not. Therefore, studies presenting solely tissue penetration ratios should be interpreted with this in mind.

Most accepted clinical PK/PD targets apply to the antibiotic being either time‐or concentration‐dependent efficient in relation to a relevant MIC value. Irrespectively, it remains to be determined how long exposure is needed, both in prophylactic and treatment settings. In general, all antibiotics perform on active bacterial cell division. Thus, treatment success cannot be achieved on dormant bacteria, which further challenges the understanding of when the antibiotic actually should work. For example, it remains uncertain whether concentration‐dependent efficacy is sufficient if the peak drug concentration only surpasses the applied target, or if a specific duration of sustained peak drug concentrations is needed to encompass insufficiency against bacterial dormancy. Furthermore, it is essential to discuss the importance of any tissue‐ or anatomical‐related distribution differences (e.g. bone and soft tissue) in attaining proper target site protection or eradication. This includes assessment of tissue vitality, vascularization and the impact of the biochemical and physiological microenvironment. Obviously, many more questions for discussion do arise, but it is outside the scope of this review to try to answer these. We hope that the proposed results can contribute valuable knowledge that can help define relevant systemic dosing regimens and guide future study designs.

### Perspectives and limitations

Several studies suggest that tissue pharmacokinetics in critically ill and sepsis patients are fundamentally different from healthy volunteers [123, 124, 125, 126]. Tissue distribution is believed to be compromised as inflammation, changes in interstitial volume, augmented renal clearance and blood constitution changes the systemic circulation, which can lead to therapeutic failure [123, 124, 125, 126, 127, 128, 129, 130]. It is evident from this review that these changes can apply to varying degrees in patients with trauma, diabetes and/or orthopaedic infections. Most of the included studies contained data from healthy tissues. Indeed, there is a pressing call for additional antibiotic PK studies in therapeutic scenarios.

In total, 43% of the included studies utilized animal models with inherent translational limitations. Interpreting results across rodents and large animal models can be complex due to the substantial interspecies differences. Pigs were the most employed model (34% of all studies). The anatomical and physiological similarities between pigs and humans have positioned pigs as robust models for pharmacokinetic studies, particularly because clinically relevant antibiotic dosing regimens can be applied [131, 132].

While dynamic sampling can yield a rich dataset, sample sizes in antibiotic PK studies are often small. To maximize the value of this data, employing population PK/PD models can be beneficial. These models allow for simulating alternative dosing regimens and modes of administration, enabling the assessment of the probability of reaching a relevant target. Undoubtedly, there is potential for the introduction of more population PK/PD models in orthopedically relevant settings.

Only two databases were searched, and only English articles were included, leaving room for possible bias in the selected articles.

## CONCLUSION

This review presents valuable insights into the microenvironmental distribution of antibiotics in orthopedically relevant target tissues and settings and seeks to provide a basis for improving dosing recommendations and treatment outcomes. However, it is important to acknowledge that our findings are limited to the specific drug, dosing regimens, administration method and target tissue and are crucially linked to the selected PK/PD target. The multifactorial influence of the microenvironment urges further research to address the significant knowledge gaps related to other antibiotic drugs and in more orthopedically relevant settings.

## Data Availability

Data sharing is not applicable to this article as no new data were created or analyzed in this study.
